# Construction of the reduced nicotinamide adenine dinucleotide salvage pathway in artificial cells and its application in amino acid synthesis†

**DOI:** 10.1039/d5sc00852b

**Published:** 2025-06-06

**Authors:** Yiming Liu, Shanshan Du, Xiangxiang Zhang, Chao Li, Shubin Li, Wenxia Xu, Jingjing Zhao, Wei Mu, Xiaojun Han

**Affiliations:** a State Key Laboratory of Urban Water Resource and Environment, MIIT Key Laboratory of Critical Materials Technology for New Energy Conversion and Storage, School of Chemistry and Chemical Engineering, Harbin Institute of Technology Harbin 150001 China hanxiaojun@hit.edu.cn lishubin@hit.edu.cn zhaojingjing@hit.edu.cn muwei@hit.edu.cn

## Abstract

Reduced nicotinamide adenine dinucleotide (NADH) salvage pathway reconstitution is a crucial step toward autonomous artificial cells. In living systems, d-ribose is a fundamental precursor intricately involved in the synthesis of nucleotides, and other critical metabolic pathways. An NADH synthesis pathway in artificial cells starting from d-ribose was constructed with a five-enzyme cascade containing ribokinase, ribose-phosphate pyrophosphokinase, nicotinamide phosphoribosyltransferase, nicotinamide mononucleotide adenylyltransferase, and formate dehydrogenase (RK, RPPK, NAMPT, NMNAT, and FDH), which efficiently converted 10 mM d-ribose into 415 μM NADH within 80 minutes under optimized conditions. The produced NADH was further used to drive the amino acid metabolism, *i.e.*, to convert NH_4_^+^ and α-ketoglutarate to glutamate by introducing additional glutamate dehydrogenase (GDH) inside artificial cells. The successful reconstitution of the NADH synthesis pathway lays the foundation for fabricating artificial cells with complicated metabolic networks.

## Abstract 2

An artificial cell capable of producing NADH from d-ribose *via* the salvage pathway was constructed by encapsulating five enzymes. The synthesized NADH was utilized for glutamate biosynthesis.

## Graphical abstract

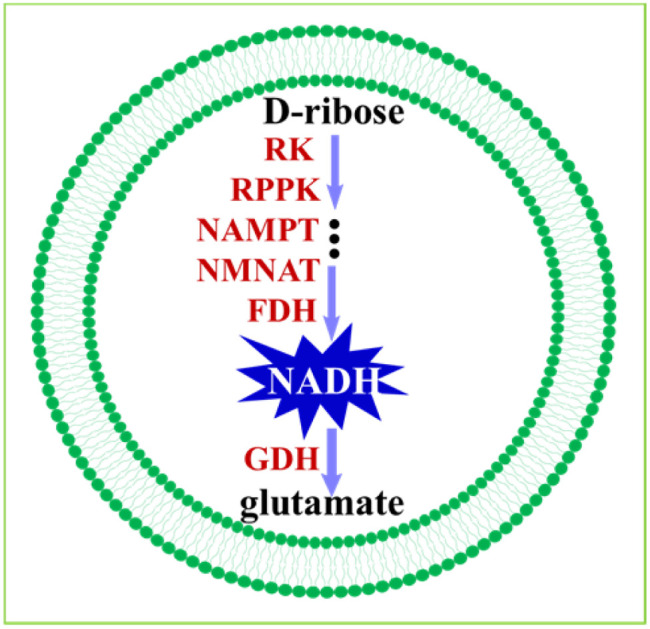

## 1. Introduction

Reduced nicotinamide adenine dinucleotide (NADH) is a critical cofactor in living systems, playing a central role in electron transfer and redox balance in metabolic reactions.^1–3^ It is indispensable for cellular energy generation and biosynthetic processes, supporting key metabolic activities to sustain life.^4,5^ Beyond its metabolic functions, NADH also contributes to maintaining cellular homeostasis through its involvement in stress responses and signaling pathways.^6,7^ Due to its essential role across various biological processes, an efficient and stable supply of NADH has become a key challenge in the field of artificial cells.

The building of artificial cells from scratches helps to understand the working mechanism of cells and provides new insights into the origins of life.^8,9^ These synthetic systems are designed to replicate fundamental cellular functions, including metabolism,^10–12^ signal transduction,^13,14^ and cell division.^15–17^ In amino acid biosynthesis, the glycine was derived by a carbon fixation metabolic cycle for the further synthesis of proteins.^18^ The establishment of complex functional metabolic pathways in artificial cells remains challenging, with one key limiting factor being the maintenance of a stable supply of electron carriers such as NADH, which are essential for driving metabolic reactions.^19^

Currently, NADH synthesis primarily relied on simple chemical or enzymatic systems.^20^ Chemical synthesis, such as photocatalysis and electrocatalysis, showed promise but faced challenges of poor biocompatibility, high energy demand, and low selectivity, hindering their integration into artificial cells.^21,22^ For instance, photocatalytic NAD^+^ reduction using ZnTPPS and [Cp*Rh(bpy)(H_2_O)]^2+^ generated various inactive isomers and dimers.^23^ Similarly, electrocatalytic methods required high overpotentials and often suffered from the formation of byproducts like 1,6-NADH.^24^ Enzymatic systems often utilized glucose dehydrogenase (GluDH) or lactate dehydrogenase (LDH) to regenerate NADH from NAD^+^. For example, NADH regeneration was driven by lactate dehydrogenase for glutamate synthesis from α-ketoglutarate and ammonium in the cell-free systems.^25^ These processes necessitated constant substrate replenishment, limiting their compatibility with self-sustaining artificial cells.^12,20,26,27^ These limitations underscore the need for innovative approaches to enable efficient NADH synthesis and integration into artificial cells.

In living organisms, NADH synthesis involves complex multi-enzyme cascades starting from tryptophan.^28,29^ The reconstitution of such a complex biosynthetic pathway *in vitro* is evidently highly challenging. As an alternative, d-ribose is a biologically relevant and cost-effective precursor for NADH synthesis, providing an ideal starting point for constructing a simplified salvage pathway.^30^d-Ribose is a precursor of 5-phosphoribosyl-1-pyrophosphate (PRPP) and participates in nucleotide metabolism, offering essential intermediates for redox reactions.^31,32^ Moreover, its significance in the “RNA World” hypothesis implies its evolutionary relevance.^33^

Herein, we constructed a salvage pathway for NADH biosynthesis inside artificial cells using a five-enzyme cascade starting from d-ribose. This system achieved efficient NADH synthesis and was further integrated with glutamate production, linking nucleotide and amino acid metabolism. The established NADH synthesis pathway provides the key nodes of natural metabolic networks, laying a strong foundation for reconstructing complex metabolic processes in artificial cells.

## 2. Results and discussion

**Fig. 1:**
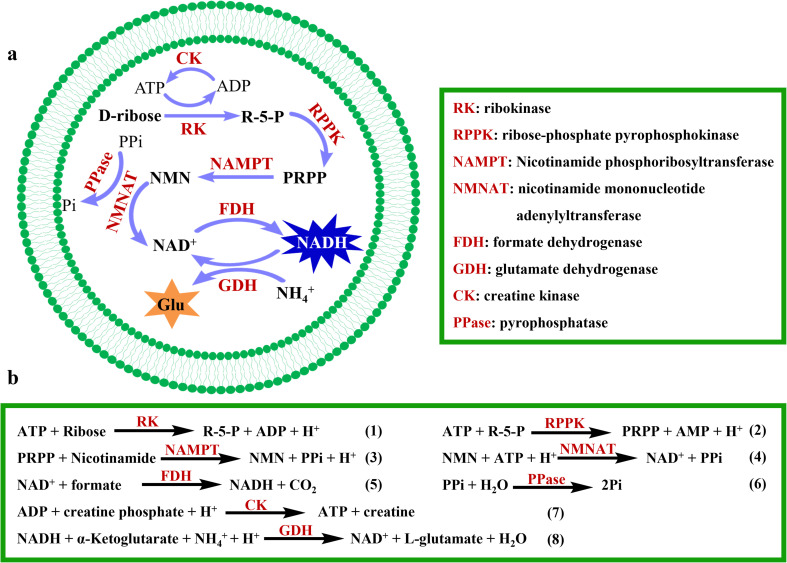
Schematic of the NADH salvage synthesis pathway within artificial cells. (a) Diagram of an artificial cell containing a metabolic pathway converting d-ribose to NADH and the further amino acid conversion pathway. (b) Reaction equations in the pathway.

The concept of this study is illustrated in Fig. 1. The metabolic pathway converting d-ribose to NADH has been reconstituted inside giant unilamellar vesicles (GUVs). The enzymes involved in the pathway are ribokinase (RK, EC:2.7.1.15), ribose-phosphate pyrophosphokinase (RPPK, EC:2.7.6.1), nicotinamide phosphoribosyltransferase (NAMPT, EC:2.4.2.12), nicotinamide mononucleotide adenylyltransferase (NMNAT, EC:2.7.7.18), and formate dehydrogenase (FDH, EC:1.17.1.9). The obtained NADH is further used to participate in an amino acid metabolic pathway from NH_4_^+^ to glutamate.

In the pathway, R-5-P is synthesized from d-ribose and ATP catalyzed by RK, producing one molecule of ADP (eqn (1), Fig. 1b). R-5-P and ATP are then converted into PRPP and AMP through the catalytic activity of RPPK (eqn (2), Fig. 1b). PRPP and NAM are further transformed into NMN by NAMPT (eqn (3), Fig. 1b). Subsequently, NMN and ATP are converted into NAD^+^, catalyzed by NMNAT (eqn (4), Fig. 1b). Finally, NAD^+^ is reduced to NADH by FDH, accompanied by the generation of CO_2_ (eqn (5), Fig. 1b). To investigate the feasibility of NADH production *in vitro*, the standard Gibbs energy changes (Δ*G*′°) of the reactions were calculated at pH 8.0 (Fig. S1†). The Δ*G*′° decreases significantly at each step, with the cumulative total Δ*G*′° of the five-enzyme cascade being −64.8 kJ mol^−1^, indicating that this process is thermally favorable.

To enhance the production yield of NADH, CK regenerates ATP from ADP and creatine phosphate (CP) to recycle ATP; meanwhile, PPase hydrolyzes PPi into inorganic phosphate (Pi) to facilitate the reactions (eqn (6) and (7), Fig. 1b). The synthesized NADH is subsequently utilized for NH_4_^+^ assimilation, producing glutamate under the catalysis of glutamate dehydrogenase (GDH), thereby integrating the pathway with amino acid metabolism (eqn (8), Fig. 1b). This successful coupling of NADH production with downstream metabolic pathways lays the foundation for building the more sophisticated artificial cells.

### 2.1 Synthesis of NMN

**Fig. 2:**
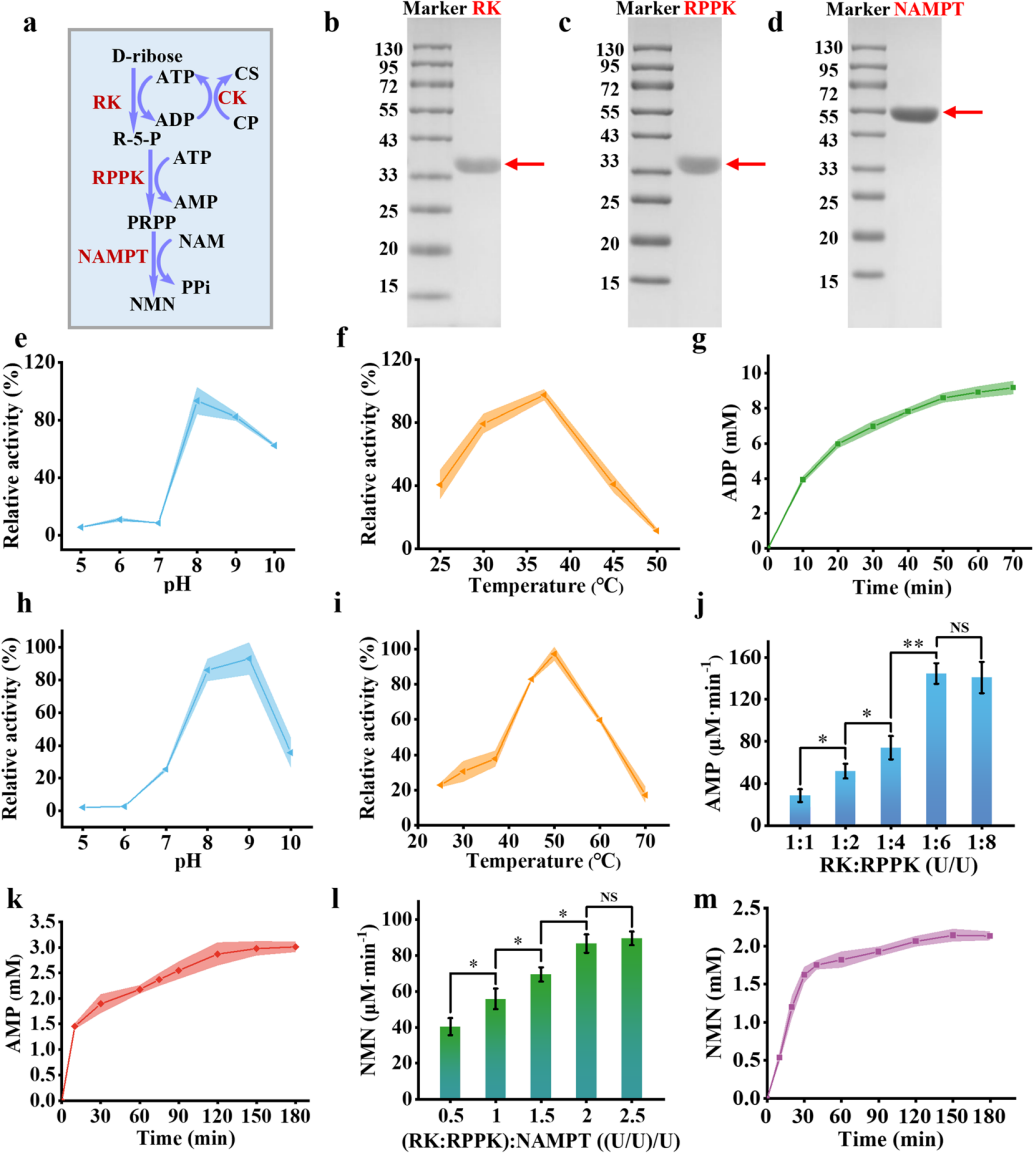
Synthesis of NMN. (a) Schematic illustration of the enzymatic pathway for converting d-ribose to NMN in solution. (b)–(d) SDS-PAGE analysis of the key enzymes in the pathway: RK from *E. coli* (34 kDa) (b), RPPK from *M. tuberculosis* (35 kDa) (c), and NAMPT from *C. pinensis* (55 kDa) (d). Influence of pH (e) and temperature (f) on the enzymatic activity of RK. (g) Time-course analysis of ADP formation in a solution containing d-ribose (10 mM), ATP (10 mM), and RK (300 U mL^−1^) at 37 °C in Tris–HCl buffer (pH 8.0). Effect of pH (h) and temperature (i) on RPPK activity. (j) The production rate of AMP at varying ratios of RK and RPPK enzyme units, with RPPK concentrations ranging from 300 to 1800 U mL^−1^ and a fixed RK concentration of 300 U mL^−1^ at 37 °C in Tris–HCl buffer (pH 8.0). (k) Time-course analysis of AMP formation over time in a solution containing d-ribose (10 mM), ATP (10 mM), sodium creatine phosphate (10 mM), CK (60 μg mL^−1^), RK (300 U mL^−1^), and RPPK (1800 U mL^−1^) at 37 °C in Tris–HCl buffer (pH 8.0). (l) The production rate of NMN at varying NAMPT concentrations (150–750 U mL^−1^) from d-ribose in a solution containing 300 U mL^−1^ RK and 1800 U mL^−1^ RPPK at 37 °C in Tris–HCl buffer (pH 8.0). (m) Time-course analysis of NMN synthesis in a solution containing d-ribose (10 mM), ATP (10 mM), sodium creatine phosphate (10 mM), NAM (5 mM), CK (60 μg mL^−1^), RK (300 U mL^−1^), RPPK (1800 U mL^−1^), and NMNAT (600 U mL^−1^) at 37 °C in Tris–HCl buffer (pH 8.0). Error bars represent the mean ± standard deviation (SD) from three independent experiments (*n* = 3). Statistical significance was assessed using a two-tailed unpaired Student's *t*-test. Statistical thresholds are denoted as *P* < 0.05, *P* < 0.01, and *P < 0.001. Differences with *P* ≥ 0.05 were considered non-significant (NS).

Nicotinamide Mononucleotide (NMN) serves as a critical precursor for NADH biosynthesis.^34^ Here, we use a three-step enzymatic metabolic reaction to synthesize NMN from d-ribose. One molecule of d-ribose was phosphorylated by RK to yield R-5-P and ADP. Subsequently, R-5-P undergoes further phosphorylation to generate PRPP, resulting in the formation of one molecule of AMP. PRPP then reacted with NAM to produce NMN, accompanied by the release of PPi (Fig. 2a). We purified and characterized those three enzymes involved in the cascade process. RK from *Escherichia coli* showed an apparent molecular weight of just over 33 kDa (Fig. 2b), closely aligning with the theoretical molecular weight of 34 kDa.^35^ RPPK from *Mycobacterium tuberculosis* showed an apparent molecular weight between 33 and 43 kDa (Fig. 2c), consistent with previously reported data of approximately 35 kDa.^36^ NAMPT from *Chitinophaga pinensis* showed an apparent molecular weight of approximately 55 kDa (Fig. 2d), similar to its theoretical value of 55 kDa.^30^

To investigate the properties of RK, we examined its optimal pH and optimal temperature. RK exhibited optimal activity at pH 8 (Fig. 2e) and 37 °C (Fig. 2f), as determined by ADP production analyzed *via* high-performance liquid chromatography (HPLC). RK catalyzed the phosphorylation of d-ribose, with an apparent Michaelis constant of 0.6686 mM, reflecting a strong affinity between RK and d-ribose (Fig. S2†). Under optimal conditions, the ribose phosphorylation reaction reached equilibrium within 70 minutes, achieving an equilibrium conversion rate of approximately 90.4% (Fig. 2g). RPPK exhibited optimal activity at pH 9 (Fig. 2h) and 50 °C (Fig. 2i), as determined by AMP production using HPLC. Notably, RPPK functioned as a phosphate-activated enzyme, exhibiting activity only in the presence of Pi. Under the conditions tested, maximum activity was achieved at a Pi concentration of 40 mM (Fig. S3†). Consequently, 40 mM KH_2_PO_4_ was incorporated into the buffer for subsequent enzyme activity assays. Furthermore, ADP was found to significantly inhibit RPPK activity,^37^ with the inhibition becoming increasingly pronounced as the ADP concentration increased (Fig. S4†). Subsequently, we will cascade RK and RPPK for an enzymatic cascade assay. In the cascade reaction, the production of ADP from the first reaction necessitated its conversion to ATP to enhance RPPK activity. CK, utilizing creatine phosphate (CP) as its substrate, plays a pivotal role in this conversion. The hydrolysis of CP, with a standard free energy of −43.1 kJ mol^−1^, is significantly more exergonic than that of ATP (−30.6 kJ mol^−1^), making the reaction highly efficient. Moreover, CK's low Michaelis constant (*K*_m_) for ADP enables efficient ATP regeneration even at low ADP concentrations, thereby maintaining minimal ADP levels and ensuring sustained catalytic efficiency.^38^ HPLC measurements showed that CK can reach reaction equilibrium within one minute, demonstrating extremely high catalytic efficiency (Fig. S5†). Consequently, CK was chosen to effectively lower ADP concentration in the system.

At pH 8 and 37 °C, varying amounts of RPPK (ranging from 300 U mL^−1^ to 2100 U mL^−1^) were combined with a fixed concentration of RK (300 U mL^−1^) to produce PRPP. The AMP production rate increased from 11.63 μM min^−1^ to 31.40 μM min^−1^ as the RPPK concentration was increased from 300 U mL^−1^ to 1800 U mL^−1^ and levelled off afterwards (Fig. 2j). Based on these results, the optimal enzyme concentrations for the subsequent cascade reactions were determined to be 300 U mL^−1^ for RK and 1800 U mL^−1^ for RPPK. Under these conditions, 10 mM d-ribose reached equilibrium within 120 minutes, yielding approximately 2.98 mM AMP with the conversion rate being approximately 29.8% (Fig. 2k). Due to the prohibitively high cost of PRPP, an enzyme cascade was employed to synthesize PRPP for assessing NAMPT activity, with NMN levels quantified by HPLC. Under the same conditions, NAMPT was subsequently introduced to facilitate NMN production. As the NAMPT loading increased from 150 U mL^−1^ to 750 U mL^−1^, the NMN production rate increased from 40.41 μM min^−1^ to 86.62 μM min^−1^ (Fig. 2l). Based on these results, a NAMPT loading of 600 U mL^−1^ was selected for subsequent enzyme cascade reactions. Under these conditions, the reaction reached equilibrium within 120 minutes, yielding approximately 2.14 mM NMN, with a conversion rate of approximately 21.4% (Fig. 2m). The successful syntheses of NMN from d-ribose were demonstrated in the solution for the subsequent enzymatic reactions to produce NADH.

### 2.2 Synthesis of NADH

**Fig. 3:**
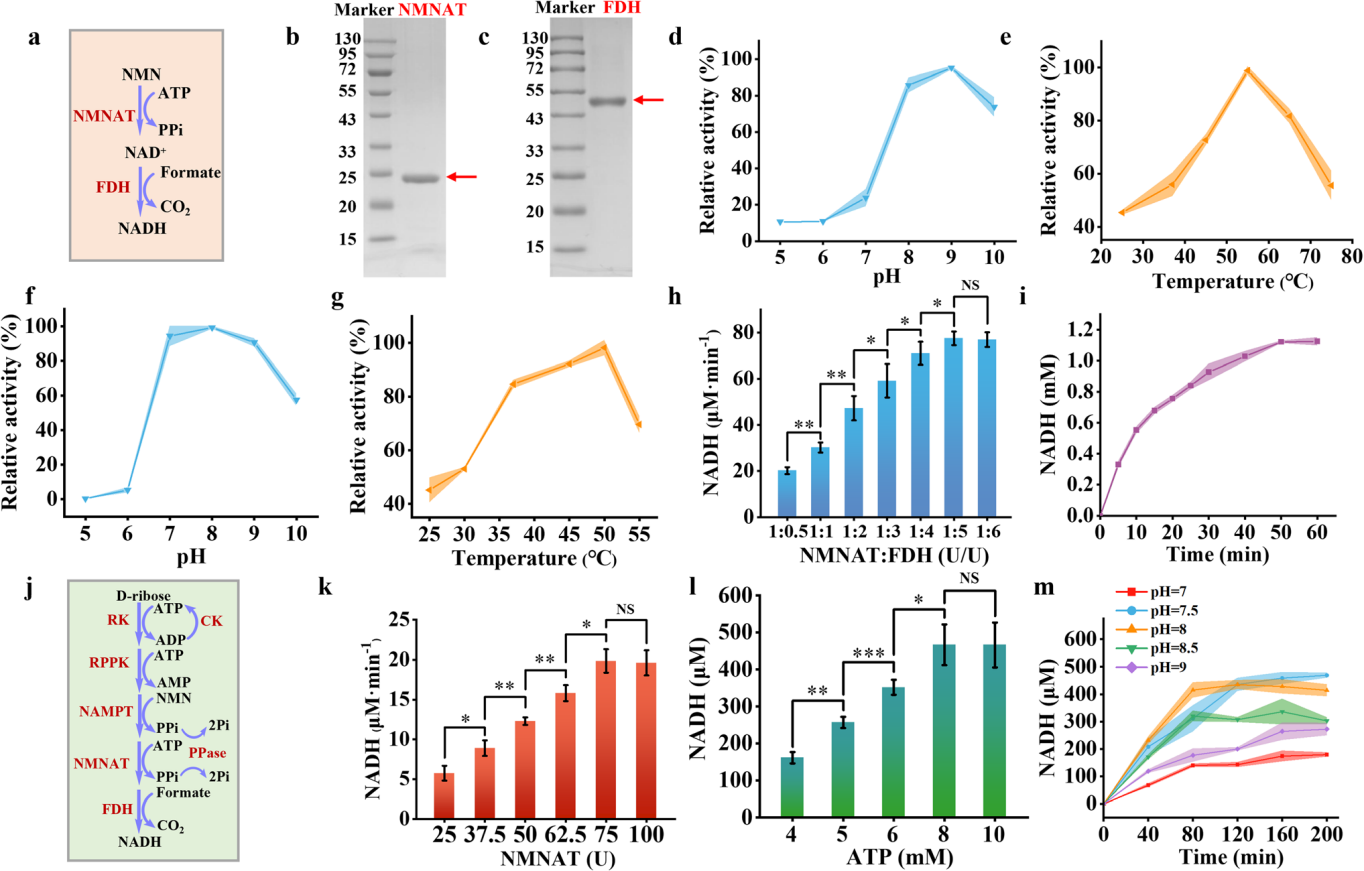
Synthesis of NADH. (a) Schematic representation of the enzymatic reaction converting NMN to NADH in solution. (b) and (c) SDS-PAGE analysis of NMNAT from *A. baylyi* (22 kDa) (b) and FDH from *M. tuberculosis* (44 kDa) (c). Effect of pH (d) and temperature (e) on the enzymatic activity of NMNAT. Effect of pH (f) and temperature (g) on the activity of FDH. (h) The rate of NADH formation at varying ratios of NMNAT and FDH from NMN, with FDH concentrations ranging from 25 to 300 U mL^−1^ at a fixed NMNAT concentration of 50 U mL^−1^ at 37 °C in Tris–HCl buffer (pH 8.0). (i) Time-course analysis of NADH concentration in a solution containing NMN (2 mM), ATP (2 mM), sodium formate (2.4 mM), NMNAT (50 U mL^−1^), and FDH (250 U mL^−1^) at 37 °C in Tris–HCl buffer (pH 8.0). (j) Schematic illustration of the complete reaction pathway from d-ribose to NADH in solution. (k) The rate of NADH formation at varying loading amount of NMNAT and FDH at a fixed ratio (1 : 5) with RK (300 U mL^−1^), RPPK (1800 U mL^−1^), and NAMPT (600 U mL^−1^) at 37 °C in Tris–HCl buffer (pH 8.0). (l) NADH production at varying ATP concentrations (4–10 mM) under reaction conditions containing RK (300 U mL^−1^), RPPK (1800 U mL^−1^), NAMPT (600 U mL^−1^), NMNAT (75 U mL^−1^), and FDH (375 U mL^−1^) at 37 °C in Tris–HCl buffer (pH 8.0). (m) Time-course analysis of NADH production in a solution containing d-ribose (10 mM), NAM (5 mM), ATP (8 mM), sodium formate (2.4 mM), sodium creatine phosphate (10 mM), RK (300 U mL^−1^), RPPK (1800 U mL^−1^), NAMPT (600 U mL^−1^), NMNAT (75 U mL^−1^), and FDH (375 U mL^−1^) at 37 °C across different pH conditions. Colored bands and error bars indicate mean ± standard deviation (SD) from three independent experiments (*n* = 3). Statistical significance was assessed using a two-tailed unpaired Student's *t*-test, with *P < 0.05, *P  < 0.01, and **P  < 0.001 indicating varying levels of significance. A *P*-value <0.05 was considered statistically significant, while NS denotes non-significant differences.

The NADH salvage pathway was established by converting NMN into NAD^+^ and subsequently reducing it to NADH. This process involved a two-step enzymatic cascade, *i.e.*, NMNAT catalyzing the reaction between NMN and ATP to produce NAD^+^ and pyrophosphate (PPi), subsequently FDH catalyzing the reduction of NAD^+^ to NADH using sodium formate as a substrate (Fig. 3a). We successfully purified and characterized NMNAT from *Acinetobacter baylyi* and FDH from *Ancylobacter novellus*. SDS-PAGE analysis revealed that the NMNAT exhibited an apparent molecular weight between 20 and 25 kDa (Fig. 3b), aligning with the theoretical molecular weight (22 kDa).^39^ FDH showed a molecular weight of just over 43 kDa (Fig. 3c), which closely matched with the theoretical estimate of 44 kDa.^40^

Due to the limited peak area of NAD^+^ observed in HPLC analysis (Fig. S6†), NMNAT activity was assessed by monitoring the depletion of its substrate of NMN. NMNAT exhibited optimal enzymatic activity at pH 9 (Fig. 3d) and 55 °C (Fig. 3e). Under mildly acidic conditions (pH 8.0), the enzyme retained 85.82% of its maximal activity. At physiological temperature (37 °C), it maintained 55.89% of its peak activity. Kinetic analysis revealed a Michaelis constant of 0.3966 ± 0.0596 mM NMNAT for the NMN substrate, indicating that NMNAT possesses a strong affinity for NMN (Fig. S7†). FDH exhibited optimal activity at pH 8 (Fig. 3f) and 50 °C (Fig. 3g), which was measured by the production of NADH with the absorbance at 340 nm. It retained 84.72% of its maximum activity at 37 °C. The Michaelis constant of FDH was 1.7286 ± 0.29 mM for sodium formate (Fig. S8†).

Overall, the enzymes involved in the NADH salvage pathway exhibited optimal performance under alkaline conditions and maintained substantial activity at 37 °C. At pH 8 and 37 °C, varying concentrations of FDH (ranging from 25 U mL^−1^ to 250 U mL^−1^) were combined with a fixed amount of NMNAT (50 U mL^−1^) to produce NADH. The NADH production rate increased from 20.13 μM min^−1^ to 77.50 μM min^−1^ as the FDH concentration was raised from 25 U mL^−1^ to 250 U mL^−1^ before reaching a plateau (Fig. 3h). For the optimized enzyme cascade, the enzyme unit ratio of NMNAT to FDH was set at 1 : 5. Under these conditions, 2 mM NMN reached equilibrium within 100 minutes, yielding approximately 1.12 mM NADH with the conversion rate of approximately 56% (Fig. 3i).

We integrated all enzymes participating in NADH synthesis, including RK, RPPK, NAMPT, NMNAT, and FDH (Fig. 3j). The loading amounts of RK, RPPK, and NAMPT were fixed based on their initial ratios, while the loading amounts of NMNAT and FDH were adjusted proportionally to optimize NADH production. The NADH production rate increased from 5.74 μM min^−1^ to 19.85 μM min^−1^ as the NMNAT concentration was elevated from 25 U mL^−1^ to 75 U mL^−1^, plateauing thereafter (Fig. 3k). Therefore, NMNAT and FDH were set at 75 U mL^−1^ and 375 U mL^−1^ for the whole metabolic pathway, respectively. Notably, ATP played a pivotal role in the enzymatic cascade for NADH synthesis, as three steps in the pathway consume ATP. Enhancing ATP concentrations from 4 mM to 10 mM significantly improved NADH yield, reaching equilibrium at concentrations above 8 mM (Fig. 3l). Elevated ATP concentrations did not further enhance NADH yield within 2 hours, likely due to excessive ADP accumulation caused by the higher initial ATP levels, which inhibited the catalytic efficiency of RPPK. Since PPi was generated in two reaction steps, we introduced pyrophosphatase to hydrolyze PPi, mitigating its inhibitory effects on the enzymatic reactions and increasing NADH yield by approximately 8.20% (Fig. S9a†). Following comprehensive optimization of reaction conditions, the pathway approached equilibrium at pH 8.0 and 37 °C within 80 minutes, achieving a final NADH concentration of approximately 415 μM (Fig. 3m). Without the addition of the ATP regeneration system (CK and CP), almost no NADH was produced, indicating the important role of the ATP regeneration system (Fig. S9b†). The NADH yield was lower than that of the NADH regeneration catalyzed by a single enzyme of glucose dehydrogenase,^41^ since 5 enzymes were involved in synthesizing NADH from d-ribose to mimic natural salvage NADH synthesis pathway. Moreover, given that the alkaline condition may influence on the efficiency of the CK/CP-based ATP regeneration system, the alternative ATP regeneration system such as polyphosphate kinase (PPK) or acetate kinase (ACK) could be involved in improving NADH production.^42^ By evaluating this process under varying pH conditions (Fig. 3m), the production rate was highest at pH 8.0 with a similar conversion rate at pH 7.5. Therefore pH 8.0 was chosen for NADH production. It was ready to encapsulate the NADH producing metabolic pathway inside artificial cells.

### 2.3 Construction of the NADH synthesis metabolic pathway in artificial cells

Before encapsulating the NADH synthesis metabolic pathway, we assessed the stability of the vesicles. GUVs composed of POPC exhibited leakage rates of 4.06% after 2 hours of incubation at 37 °C, demonstrating that they remained largely stable in the 2-hour period and were suitable for constructing the NADH metabolic pathway (Fig. S10†). Furthermore, representative fluorescence images of GUVs containing calcein after incubation at 37 °C confirmed the thermal stability of the vesicles (Fig. S11†). Since NADH molecules emit blue fluorescence at 469 nm,^43^ the production of NADH can be observed visually by fluorescence microscopy. To confirm this, the conversion of NAD^+^ to NADH was conducted within artificial cells (Fig. S12a†) by encapsulating NAD^+^ (0.5 mM) and FDH (50 U mL^−1^). After adding formate into an artificial cell solution (pH = 5.0), the blue fluorescence intensity increased gradually as a function of time due to the formation of formic acid at a pH of 5.0 (Fig. S12b†), which entered artificial cells by diffusion. FocA as a formate transporter could be functionally reconstituted into the GUV membrane to allow formate uptake at neutral or alkaline pH.^44^ These results confirmed the successful encapsulation of NAD^+^ and FDH within the artificial cells and the observation of NADH production by fluorescence microscopy.

**Fig. 4:**
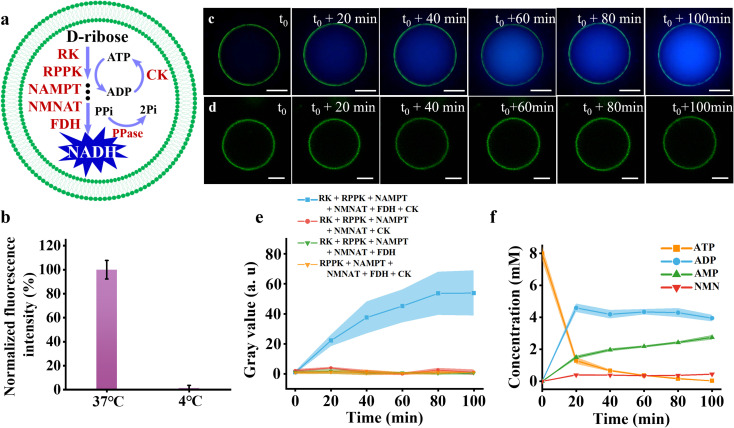
Construction of the NADH synthesis metabolic pathway in artificial cells. (a) Schematic representation of the engineered metabolic pathway for the conversion of d-ribose to NADH within artificial cells. (b) Relative fluorescence intensity of artificial cells containing RK (300 U mL^−1^), RPPK (1800 U mL^−1^), NAMPT (600 U mL^−1^), NMNAT (75 U mL^−1^), FDH (375 U mL^−1^), CK (60 μg mL^−1^), PPase (50 U mL^−1^), d-ribose (10 mM), NAM (5 mM), ATP (8 mM), sodium formate (2.4 mM), and sodium creatine phosphate (10 mM) at Tris–HCl buffer (pH 8.0) at 37 °C and 4 °C for 100 minutes. (c) Representative time-lapse fluorescence microscopy images of artificial cells incubated under the same conditions at 37 °C. (d) Time-lapse images of artificial cells similar to those in (c), but without FDH as a control. (e) Time-dependent fluorescence intensity measurements of the artificial cells encapsulating the whole pathway (blue curve), the incomplete pathway lacking FDH (red curve), the incomplete pathway lacking CK (green curve), and the incomplete pathway lacking RK (orange curve). (f) The concentration profiles of ATP (orange curve), ADP (blue curve), AMP (green curve), and NMN (red curve) within artificial cells as a function of time. The colored bands and error bars represent the mean ± standard deviation (SD) from three independent replicates (*n* = 3). Scale bars in panels (b) and (c) are 10 μm.

The complete NADH synthesis pathway was encapsulated inside artificial cells to produce NADH. Optimized enzymes (RK, RPPK, NAMPT, NMNAT, FDH, CK, and PPase) and substrates (d-ribose, ATP, NAM, sodium creatine phosphate, and sodium formate) were encapsulated within the artificial cells (Fig. 4a). The encapsulation efficiency for enzymes was measured to be 93.43 ± 5.15%. The size of the artificial cells was analyzed statistically, with an average particle size of approximately 13 μm (Fig. S13†). After 100 minutes of incubation at 37 °C, the strong fluorescence was observed in the artificial cells, whereas no fluorescence was observed at 4 °C (Fig. 4b), which indicated the metabolic reactions could be imitated by varying the temperature. Setting the moment when the temperature stabilized at 37 °C as *t*_0_, the blue fluorescence intensity within the artificial cells gradually increased over the time course of 100 minutes (Fig. 4c). In contrast, no noticeable fluorescence changes were observed in artificial cells lacking FDH (Fig. 4d). Time-dependent fluorescence intensity measurements revealed that the fluorescence reached its maximum at approximately 80 minutes and then levelled off (Fig. 4e, blue curve), consistent with the results in solution (Fig. 3m, orange curve). As controls, the average fluorescence intensity of the artificial cells missing key enzymes of FDH (Fig. 4e, red curve), CK (Fig. 4e, green curve), or RK (Fig. 4e, orange curve) showed no significant changes.

The fluorescence observation visually confirmed the production of NADH inside artificial cells. The intermediates of ATP, ADP, AMP and NMN inside artificial cells were monitored using HPLC (Fig. 4f). The ATP concentration declined rapidly during the first 20 minutes, as ATP was consumed in the first, second, and fourth steps of the pathway. Thereafter, as substrate levels diminished and ATP was partially regenerated by CK, the rate of ATP depletion slowed down, eventually approaching nearly 0 mM at 100 minutes (Fig. 4f, orange curve). The concentration of ADP increased sharply within the first 20 minutes, driven by the high catalytic activity of RK. Beyond this point, the ADP concentration gradually decreased, primarily due to ATP regeneration facilitated by CK (Fig. 4f, blue curve). The concentration of AMP gradually increased over 100 minutes, mirroring the production of PRPP due to their stoichiometric 1 : 1 ratio. After 20 minutes, the production rate slowed, likely due to the inhibitory effect of ADP on RPPK (Fig. 4f, green curve). The concentration of NMN increased steadily during the first 20 minutes, driven by the sustained enzymatic activity of the first three steps in the cascade. After 20 minutes, the NMN concentration plateaued as its production rate approximately balanced with its consumption rate (Fig. 4f, red curve). To test the stability of enzymes and GUVs, NADH synthesis pathway was encapsulated in the GUVs to monitor NADH production as a function of time. The NADH production slightly decreased by 3.1% after 24 hour storage (Fig. S14†). Based on the above-mentioned results, we successfully constructed the NADH synthesis metabolic pathway within the artificial cells, which can be used for further NADH-dependent metabolic pathways.

### 2.4 Amino acid synthesis within artificial cells equipped with the NADH metabolic pathway

**Fig. 5:**
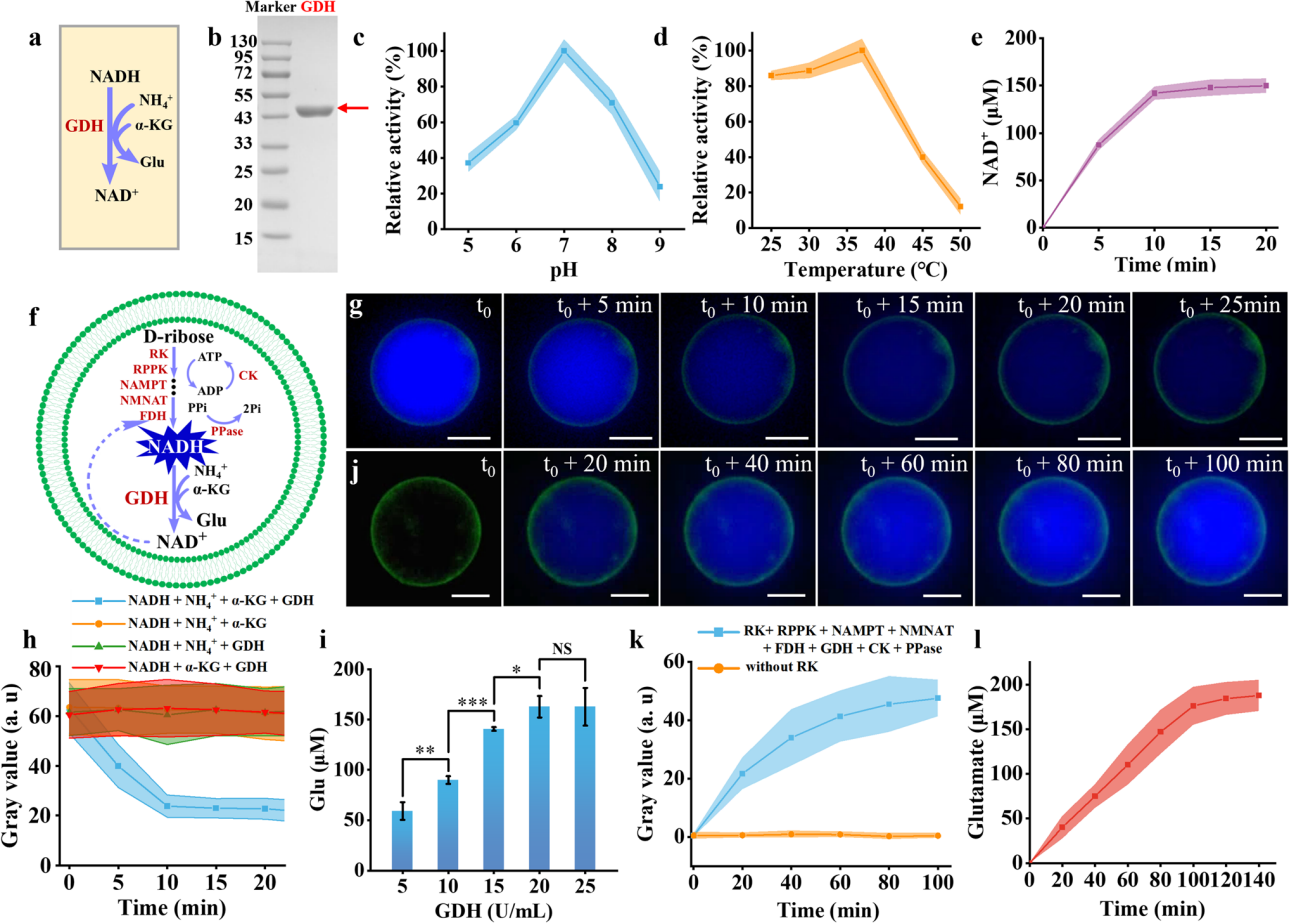
Amino acid synthesis in artificial cells equipped with the NADH metabolic pathway. (a) Schematic representation of the metabolic pathway catalyzing the conversion of NH_4_^+^ and α-ketoglutarate to glutamate. (b) SDS-PAGE gel image of GDH from *B. subtilis* (46 kDa). (c) and (d) Effect of pH (c) and temperature (d) on the enzymatic activity of GDH. (e) Time-course measurement of NAD^+^ concentration in a solution containing NAD^+^ (200 μM), NH_4_Cl (10 mM), α-ketoglutarate (400 μM), and GDH (50 U mL^−1^) at 37 °C in Tris–HCl buffer (pH 7.0). (f) Schematic representation of the integrated metabolic pathway converting d-ribose to glutamate within artificial cells. (g) Representative time-lapse fluorescence microscopy images of artificial cells containing GDH (50 U mL^−1^), 100 mM NH_4_Cl, 500 μM α-ketoglutarate, and 200 μM NADH. (h) Corresponding fluorescence intensity measurements of artificial cells in (g) over time (blue curve), as well as fluorescence intensities of control artificial cells without GDH (orange curve), α-ketoglutarate (green curve), or NH_4_Cl (red curve). (i) Production of glutamate at varying GDH concentrations (5–25 U mL^−1^) in a solution containing RK (300 U mL^−1^), RPPK (1800 U mL^−1^), NAMPT (600 U mL^−1^), NMNAT (75 U mL^−1^), FDH (375 U mL^−1^), CK (60 μg mL^−1^), PPase (50 U mL^−1^), 10 mM d-ribose, 5 mM NAM, 8 mM ATP, 2.4 mM sodium formate, 10 mM sodium creatine phosphate, 5 mM NH_4_Cl, and 500 μM α-ketoglutarate. (j) Representative time-lapse images of artificial cells containing the NADH metabolic pathway and GDH optimized at 20 U mL^−1^. (k) The corresponding fluorescence intensity in (j) (blue curve), and the control condition with no RK inside the artificial cell (orange curve). (l) Glutamate concentration in artificial cells as a function of time. The colored bands and error bars represent mean ± standard deviation (SD) from three independent experiments (*n* = 3). Statistical significance was determined using a two-tailed unpaired Student's *t*-test, with *p* < 0.05, *p* < 0.01, and *p < 0.001 indicating significance levels. A *p*-value <0.05 was considered statistically significant, while NS denotes non-significant differences. Scale bars in (g) and (j) were 20 μm.

Based on the successful construction of the NADH synthesis pathway in artificial cells, the next step was to explore its application in amino acid metabolism. Among various amino acids, glutamate was selected due to its essential role in nitrogen metabolism and its wide industrial relevance.^45^ Glutamate dehydrogenase (GDH) catalyzes ammonium ions and α-ketoglutarate (α-KG) into glutamate with the help of NADH (Fig. 5a). The GDH from *Bacillus subtilis* exhibited an apparent molecular weight exceeding 43 kDa (Fig. 5b), consistent with a theoretical value of approximately 46.58 kDa.^46^ The enzyme demonstrated maximum activity at pH 7 (Fig. 5c) and 37 °C (Fig. 5d). Under these optimal conditions, 200 μM NADH was converted into approximately 143 μM NAD^+^ (Fig. 5e). Given the stoichiometric 1 : 1 ratio between NAD^+^ and glutamate, an equivalent amount of 143 μM glutamate was simultaneously produced.

The conversion of α-KG and NH_4_^+^ to glutamate was first investigated in artificial cells containing α-KG (500 μM), NADH (0.2 mM), NH_4_Cl (10 mM), and GDH (50 U mL^−1^) (Fig. 5f). In NBD-labeled artificial cells, the blue fluorescence intensity gradually decreased and reached equilibrium after approximately 10 minutes (Fig. 5g and h). Given the stoichiometric 1 : 1 ratio between NADH consumption and glutamate production, the observed decrease in blue fluorescence within the artificial cells confirmed the synthesis of glutamate. However, in artificial cells without GDH, no significant change in blue fluorescence was observed during the same period (Fig. S15†). Analysis of the relative fluorescence intensity in artificial cells lacking NH_4_Cl (Fig. 5h, orange curve), α-KG (Fig. 5h, purple curve), or GDH (Fig. 5h, blue curve) showed no changes, confirming that all three components are essential for glutamate production.

Before coupling NADH production with glutamate synthesis, we optimized the amount of GDH in the solution to achieve the maximum yield of glutamate. Different amounts of GDH were added to a solution containing the optimized NADH metabolic pathway to obtain the highest yield of glutamate. When the GDH concentration was increased to 20 U mL^−1^, the glutamate yield plateaued (Fig. 5i). Therefore, we selected a GDH concentration of 20 U mL^−1^ for subsequent reactions. After approximately 120 minutes, glutamate production approached equilibrium, yielding about 179 μM glutamate (Fig. S16†). Approximately 45% of NADH was used for the synthesis of glutamate. The glutamate production was not increased by increasing the substrate of α-KG from 500 μM to 1500 μM (Fig. S17†), which indicated that α-KG consumption did not influence the NADH utilization. By compromising the other enzyme activities, pH of 8.0 was chosen for glutamate production, which was different with the optimum pH (7.0) for GDH. This may explain the low utilization of NADH. Mutagenesis of GDH with optimum pH of 8.0 may promote glutamate yield. At this point, the complete metabolic pathway for glutamate synthesis has been established and is ready to be encapsulated within artificial cells for further reactions.

Artificial cells containing d-ribose, ATP, sodium creatine phosphate, NAM, sodium formate, NH_4_Cl, α-KG, RK, RPPK, NAMPT, NMNAT, FDH, GDH, CK, and PPase were constructed to facilitate glutamate production. The encapsulation rate of these enzymes was determined to be 98.5 ± 1.0%. The gradual increase in blue fluorescence intensity within the artificial cells indicated the production of NADH (Fig. 5j and k). Meanwhile, glutamate production increased gradually and plateaued after 100 minutes (Fig. 5l). The glutamate concentration within the artificial cells was approximately 184.32 μM at 120 minutes (Fig. 5l). Following 24-hour incubation at 4 °C, glutamate production in artificial cells decreased by 6.8% (Fig. S18†). Under identical experimental conditions, the removal of any enzyme involved in NADH synthesis abolished significant fluorescence changes in the artificial cells. The fluorescence intensity after the removal of RK (Fig. 5k, orange curve) confirmed the absence of NADH formation. These findings confirm the successful establishment of an amino acid metabolism pathway within the artificial cells, demonstrating a significant application of the NADH synthesis metabolic pathway. In addition to amino acid synthesis, NADH can be potentially utilized to produce intermediates for chiral pharmaceuticals inside artificial cells.^47,48^ To enhance NADH yield, several strategies such as multi-enzyme assembly,^49^ scaffold proteins,^50^ fusion proteins,^51^ or formation of condensates^52^ are the potential solutions in the future.

## 3. Conclusions

The NADH metabolic pathway was built inside an artificial cell for amino acid synthesis. The NADH synthesis pathway converted d-ribose to NADH using RK, RPPK, NAMPT, NMNAT and FDH. Approximately 415 μM NADH was produced from 10 mM d-ribose within 80 minutes in the optimized experimental conditions. Coupling this pathway with glutamate synthesis achieved the integration of nucleotide and amino acid metabolism, producing 176 μM glutamate inside artificial cells. The construction of the NADH synthesis pathway inside artificial cells provides great opportunities for complicated metabolic network reconstitution inside artificial cells, further stepping forward to minimal cells. Currently the efficient harmonization of metabolic networks is still challenging in the artificial cells. The potential solutions could be using an engineered membrane transporter to balance the mass intake and exportation, establishing efficient ATP regeneration systems to guarantee a steady and long-lasting energy supply, coordinating enzyme cascades to prevent metabolic bottlenecks with fusion proteins, and implementing metabolic pathways in a spatially controlled way using subcellular microcompartments inside artificial cells.

## 4. Experimental

### 4.1 Materials

1-Palmitoyl-2-oleoyl-*glycero*-3-phosphocholine (POPC) was obtained from Avanti Polar Lipids (USA). *N*-(7-Nitrobenz-2-oxa-1,3-diazol-4-yl)-1,2-dihex-adecanoyl-*sn*-glycero-3-phosphoethanolamine triethylammonium salt (NBD-PE) was obtained from Thermo Fisher Scientific (USA). Protein markers (15–130 kDa), TE buffer (pH 8.0), kanamycin, deoxyribonuclease I (DNase I), dithiothreitol (DTT) and creatine phosphate disodium salt were obtained from Solarbio (China). A Ni-NTA 6FF prepacked chromatographic column and the plasmids were purchased from Sangon Biotech (China). Yeast extract fermentation agent, tryptone, and agar were purchased from AOBOX (China). Lysozyme, glycine, and isopropyl β-d-thiogalactoside (IPTG) were purchased from Biotopped (China). Sodium dodecylsulfate (SDS) was purchased from Gentihold (China). Trichloromethane was purchased from Kermal (China). Mineral oil was purchased from Tianjin Fuyu Fine Chemical (China). Sucrose, glucose, magnesium chloride (MgCl_2_), sodium chloride (NaCl), adenosine 5′-triphosphate disodium salt (ATP), imidazole and bovine serum albumin (BSA) were purchased from Sigma (China). Ammonium chloride (NH_4_Cl), hydrochloric acid (HCl), d-ribose, ribose-5-phosphate (R-5-P), adenosine diphosphate (ADP), adenosine monophosphate (AMP), nicotinamide (NAM), nicotinamide mononucleotide (NMN), nicotinamide adenosine dinucleotide (NAD^+^), nicotinamide adenine dinucleotide hydride (NADH), α-ketoglutaric acid, monopotassium phosphate (KH_2_PO_4_), disodium hydrogen phosphate (Na_2_HPO_4_), Coomassie blue fast staining solution, phenylisothiocyanate (PITC), glutamate (Glu), and sodium formate were purchased from Aladdin Biochemical Technology (China). Pyrophosphatase (PPase) and creatine kinase (CK) were purchased from Shanghai Beyotime Biotechnology. Millipore Milli-Q water with a resistivity of 18.2 MΩ cm was used in the experiments.

### 4.2 Construction of plasmids

The genes corresponding to RK, RPPK, NAMPT, NMNAT, FDH, and GDH are rbsK, prs, nampt, nadM, fdh, and rocG, with species sources being *Escherichia coli*, *Mycobacterium tuberculosis*, *Chitinophaga pinensis*, *Acinetobacter baylyi*, *Ancylobacter novellus*, and *Bacillus subtilis*, respectively. These genes were synthesized and subcloned into the pET28a vector by Sangon Biotech (China), resulting in the plasmids pET28a-rbsK, pET28a-prs, pET28a-nampt, pET28a-nadM, pET28a-fdh, and pET28a-rocG. All genes underwent codon optimization to enhance expression in *E. coli*. The sequences of all primers are shown in Table S1.†

### 4.3 Protein expression and purification

The BL21(DE3) strain was selected for protein expression. The recombinant strains containing the aforementioned plasmids were cultured overnight at 37 °C for 200 rpm. The overnight culture was then diluted 1 : 100 in fresh medium for scale-up cultivation. When the OD_600_ of the culture reached 0.6–0.8, IPTG was added to a final concentration of 0.2 mM to induce protein expression; meanwhile, the temperature was adjusted to 18 °C. After 16 hours of incubation, the cells were harvested by centrifugation at 4 °C for 8000 rpm. The collected cells were resuspended in Tris–HCl buffer (pH = 8.0) and lysed by sonication, followed by centrifugation at 4 °C for 12 000 rpm to collect the supernatant. The supernatant was then purified using Ni-chelating affinity chromatography, and the protein purity was assessed by SDS-PAGE, with the protein concentration determined using the Bradford assay. The calibration curve for protein concentration determination is shown in Fig. S19.†

### 4.4 Assay of RK activity

The activity of RK was measured at 37 °C by monitoring the production of ADP by using HPLC.^30^ The assay system consisted of 10 mM d-ribose, 10 mM ATP, 5 mM MgCl_2_, and 1 U mL^−1^ purified enzyme in 50 mM Tris–HCl buffer (pH 8.0). The reaction was started by the addition of RK and stopped by adding an equal volume of 1 M HCl in an ice bath. One unit of RK activity was defined as the amount of enzyme that released 1 μmol of ADP per minute. The calibration curve of ADP was shown in Fig. S20.†

### 4.5 Assay of RPPK activity

The activity of RPPK was measured at 37 °C by monitoring the production of AMP using HPLC.^36^ The assay system consisted of 5 mM R-5-P, 5 mM ATP, 5 mM MgCl_2_, 40 mM KH_2_PO_4_ and an appropriate volume of purified enzyme in 50 mM Tris–HCl buffer (pH 8.0). The reaction was started by the addition of RPPK and stopped by adding an equal volume of 1 M HCl in an ice bath. One unit of RPPK activity was defined as the amount of enzyme that released 1 μmol of AMP per minute. The calibration curve of AMP was shown in Fig. S21.†

### 4.6 Assay of NAMPT activity

The activity of NAMPT was measured at 37 °C by monitoring the production of NMN using HPLC.^30^ In the previously mentioned RPPK activity assay system, after the reaction reaches equilibrium, the concentration of PRPP is determined by measuring the concentration of AMP. An equal volume of the same buffer containing 10 mM NAM, 5 mM MgCl_2_, and an appropriate amount of NAMPT was added to initiate the reaction. One unit of NAMPT activity was defined as the amount of enzyme that released 1 μmol of NMN per minute. The calibration curve of NMN was shown in Fig. S22.†

### 4.7 Assay of NMNAT activity

The activity of NMNAT was measured at 37 °C by monitoring the decrease of NMN by using HPLC. The assay system consisted of 5 mM NMN, 5 mM ATP, 5 mM MgCl_2_, 40 mM KH_2_PO_4_ and an appropriate volume of purified enzyme in 50 mM Tris–HCl buffer (pH 8.0). The reaction was started by the addition of NMNAT and stopped by adding an equal volume of 1 M HCl in an ice bath. One unit of NNMAT activity was defined as the amount of enzyme that released 1 μmol of NAD^+^ per minute.

### 4.8 Assay of FDH activity

The activity of FDH was measured at 37 °C by monitoring the decrease of NADH at 340 nm using a UV-vis spectrometer.^40^ The assay system consisted of 200 μM NAD^+^, 400 μM sodium formate, 5 mM MgCl_2_, and an appropriate volume of purified enzyme in 50 mM Tris–HCl buffer (pH 8.0). One unit of FDH activity was defined as the amount of enzyme that released 1 μmol of NADH per minute. The calibration curve of NADH was shown in Fig. S23.†

### 4.9 Assay of GDH activity

The activity of GDH was measured at 37 °C by monitoring the decrease of NADH at 340 nm using a UV-vis spectrometer. The assay system consisted of 150 μM NADH, 100 mM NH_4_Cl, 0.5 mM α-ketoglutarate, 5 mM MgCl_2_ and an appropriate volume of purified enzyme in 50 mM Tris–HCl buffer (pH 7.0). One unit of GDH activity was defined as the amount of enzyme that released 1 μmol of NAD^+^ per minute.

### 4.10 Effect of pH and temperature on the activity of purified enzymes

The effect of pH on purified enzymes was assessed at 37 °C using buffers with varying pH levels, ranging from 4.0 to 10.0. Phosphate buffer (PB buffer) was used for pH values between 4.0 and 7.0, Tris–HCl buffer for pH 7.0 to 8.0, and glycine-NaOH buffer for pH 9.0 to 10.0, respectively. The optimal temperature for each enzyme was determined at its optimal pH, which was determined in Tris–HCl buffer. To identify the optimal temperature and pH of an enzyme, the initial reaction rates of product formation under varying conditions were calculated. The highest activity of the enzyme was set as 100% of relative activity.

### 4.11 Construction of artificial cells

Artificial cells were prepared by the emulsion transfer method as described elsewhere (Fig. S24†).^53^ Specifically, 1 mg of POPC and 0.05 mg of NBD-PE were dissolved in 100 μL of chloroform, followed by the addition of 1 mL of mineral oil. The mixture was incubated at 80 °C for 30 minutes to evaporate the chloroform and subsequently cooled to room temperature to yield lipid oil-phase solutions. To generate a water-in-oil emulsion, 300 μL of the lipid oil-phase solution and 30 μL of an aqueous solution were combined and vortexed for 30 seconds. The composition of the solution for the NADH synthesis metabolic pathway included 260 mM sucrose, 40 mM KH_2_PO_4_, 10 mM d-ribose, 8 mM ATP, 5 mM NAM, 5 mM MgCl_2_, 10 mM creatine phosphate sodium, 2.4 mM sodium formate, RK (300 U mL^−1^), RPPK (1800 U mL^−1^), NAMPT (600 U mL^−1^), NMNAT (75 U mL^−1^), FDH (375 U mL^−1^), CK (60 μg mL^−1^), PPase (50 U mL^−1^), and 50 mM Tris–HCl (pH 8). For the glutamate synthesis metabolic pathway, the aqueous solution was additionally supplemented with 10 mM NH_4_Cl, 500 μM α-ketoglutarate, and GDH (20 U mL^−1^). The water-in-oil emulsion was gently layered onto 200 μL of an isotonic solution (300 mM glucose and 40 mM KH_2_PO_4_ for the NADH synthesis pathway; 320 mM glucose and 40 mM KH_2_PO_4_ for the glutamate synthesis pathway) in a 1.5 mL tube. Centrifugation was performed at 11 000×*g* for 30 minutes at 4 °C. Giant unilamellar vesicles (GUVs) were collected from the bottom of the tube and used for subsequent experiments.

### 4.12 HPLC assay of ADP, AMP, NMN, and glutamate

The concentrations of ADP, AMP, and NMN were measured using HPLC with an AQ C18 column (4.6 × 150 mm, ChromCore). The mobile phase consisted of 50 mM monopotassium phosphate, flowing at 1 mL min^−1^. Column temperature was maintained at 30 °C with the detection at a wavelength of 270 nm. All samples were initially diluted in water, followed by filtration using a 0.22 μm hydrophilic filter membrane (Jinteng, Shanghai). A 10 μL aliquot of each sample was injected into the HPLC column for separation. Data acquisition and analysis were conducted using Dionex Chromeleon software.

The determination of glutamate was performed using the PITC derivatization method.^12^ The amino acid standard mixture or sample solution (200 μL), 0.2 M PITC acetonitrile solution (100 μL), and 1 M triethylamine acetonitrile solution (100 μL) were mixed at 25 °C for 1 h, followed by the addition of 400 μL *n*-Hexane. The mixture was vortexed, centrifuged at 10 000×*g* for 10 min, and filtered through a 0.22 μm Nylon 66 filtration membrane. The assay of glutamate was carried out with the AQ C18 column (4.6 × 150 mm, ChromCore). The mobile phase consisted of two components at Phase A that was a mixture of 0.1 M sodium acetate solution (pH 5.6) and acetonitrile in a 93 : 7 ratio, and Phase B that was a mixture of water and acetonitrile in a 1 : 4 ratio, respectively. The separation was performed at a flow rate of 1.0 mL min^−1^ using a gradient elution profile as follows: from 0 to 5.6 min with a linear gradient from 95% A to 52% A; from 5.6 to 5.8 min with a linear gradient from 52% A to 0% A; from 5.8 to 8 min with isocratic elution at 0% A. Column temperature was maintained at 40 °C with detection at a wavelength of 254 nm. The calibration curve of glutamate was shown in Fig. S25.†

### 4.13 Pre-column derivatization method of phenyl isothiocyanate (PITC)

200 μL of the reacted solution was added into a 1.5 mL tube, followed by the addition of 100 μL of 1 mol L^−1^ triethylamine in acetonitrile solution and 100 μL of 0.2 mol L^−1^ PITC solution. After 1 hour incubation, 400 μL of *n*-hexane was added with shaking vigorously for 5–10 seconds. 200 μL of the lower layer solution was mixed with 800 μL of water for HPLC analysis.

### 4.14 The encapsulation rate of enzymes in artificial cells

The encapsulation rate of enzymes in artificial cells was measured using the Bradford method. The artificial cells were prepared to contain RK, RPPK, NAMPT, NMNAT, FDH, and GDH. The encapsulation rate was defined as (*C*_0_ − *C*_t_)/*C*_0_ × 100%, where *C*_0_ and *C*_t_ were the initial concentration of enzymes and the concentration of unencapsulated enzymes. *C*_*t*_ was obtained by measuring the concentration of enzymes in the supernatant after centrifuging an artificial cell solution.

## Author contributions

Conceptualization: X. H. and Y. L.; methodology: Y. L., S. D., S. L., J. Z., W. M., and X. H.; investigation: Y. L., S. D., X. Z., C. L., W. X., and S. L.; visualization: Y. L., S. D., and J. Z.; funding acquisition: X. H.; project administration: X. H. and S. L.; supervision: X. H., J. Z., and W. M.; writing – original draft: Y. L., S. D., and J. Z.; writing – review & editing: X. H., Y. L., S. D., S. L. and W. M.

## Conflicts of interest

There are no conflicts to declare.

## Supplementary Material

SC-016-D5SC00852B-s001

## Data Availability

The data that support the findings of this study are included in the main text and ESI.† Additional data are available from the corresponding authors upon reasonable request.
