# Supplementary material for: Construction of the reduced nicotinamide adenine dinucleotide salvage pathway in artificial cells and its application in amino acid synthesis
Source: Chem Sci. 2025 Jun 6;16(28):12969–81. doi: 10.1039/d5sc00852b (PMC12168924; doi:10.1039/d5sc00852b)
Supplement: SC-016-D5SC00852B-s001 [file SC-016-D5SC00852B-s001.pdf]

Supplementary Information (SI) for Chemical Science.
This journal is © The Royal Society of Chemistry 2025

Supplementary Information

Construction of the reduced nicotinamide adenine dinucleotide salvage pathway in artificial cells and its application in amino acid synthesis

Yiming Liu#, Shanshan Du#, Xiangxiang Zhang, Chao Li, Shubin Li*, Wenxia Xu, Jingjing Zhao*, Wei Mu*, Xiaojun Han*

State Key Laboratory of Urban Water Resource and Environment, MIIT Key Laboratory of Critical Materials Technology for New Energy Conversion and Storage, School of Chemistry and Chemical Engineering, Harbin Institute of Technology, Harbin 150001, China

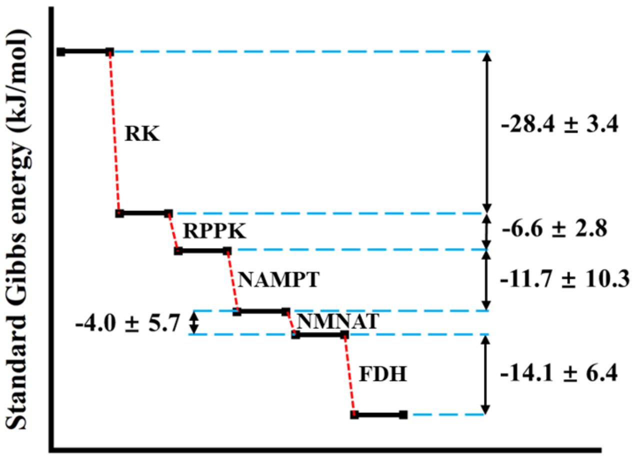

Figure S1. The standard Gibbs energy changes (ΔG'°) of NADH synthesis pathway at pH 8.0. data from http://equilibrator.weizmann.ac.il/.

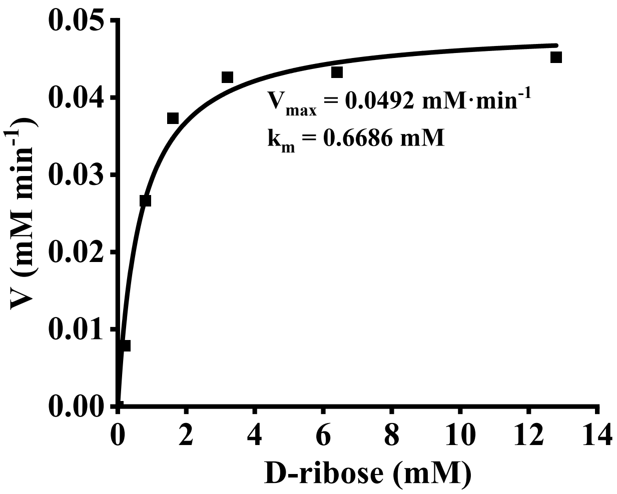

Figure S2. The initial velocities (5 mM ATP, 37°C, pH 8) of ADP as a function of D-ribose concentration.

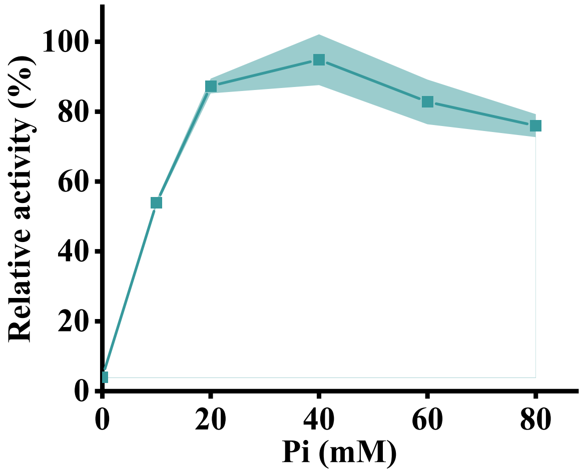

Figure S3. Relative RPPK activity at varying concentrations of inorganic phosphate (Pi) from 0 to 80 mM in 50 mM Tris-HCl (pH 8.0).

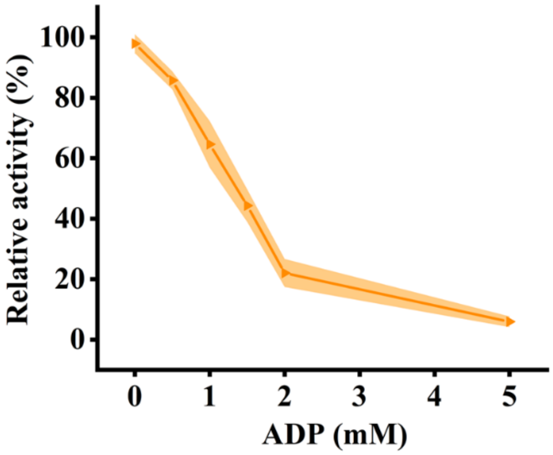

Figure S4. Relative RPPK activity at varying concentrations of ADP from 0 to 5 mM in 50 mM Tris-HCl (pH 8.0).

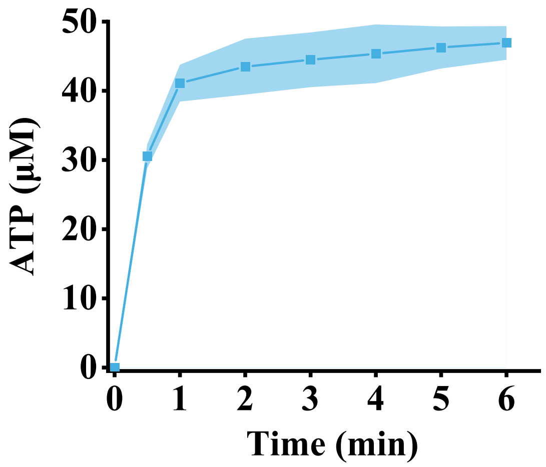

Figure S5. Concentration of produced ATP as a function of reaction time in a solution containing 60 μg/mL CK at 37°C in 50 mM Tris-HCl (pH 8.0).

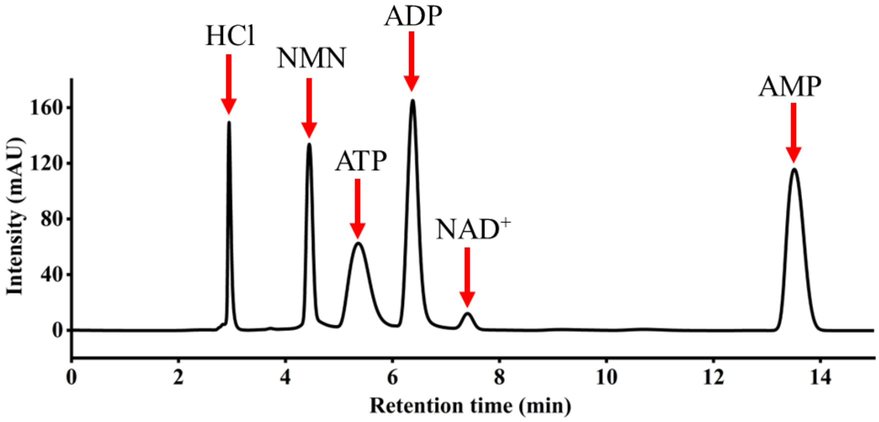

Figure S6. HPLC chromatogram of HCl (1 M), NMN (200 μM), ATP (200 μM), ADP (200 μM), NAD^+ (200 μM), and AMP (200 μM).

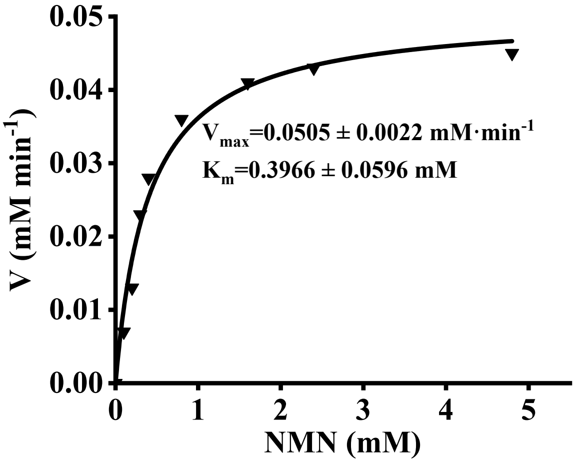

Figure S7. The initial velocities (ATP concentration of 5 mM, 37°C, pH 8.0) of NAD^+ as a function of NMN concentration.

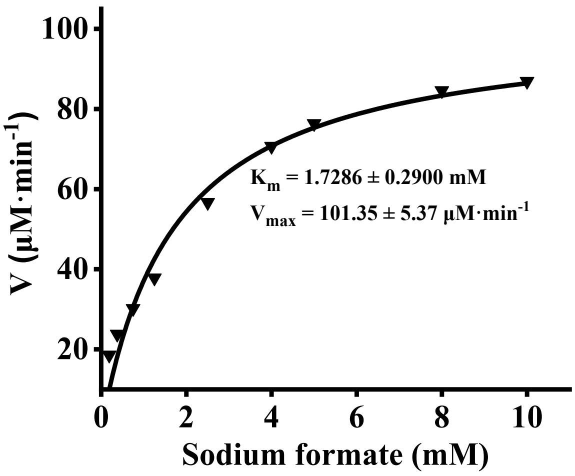

Figure S8. The initial velocities (NAD^+ concentration of 5 mM, 37°C, pH 8) of NADH as a function of sodium formate concentration.

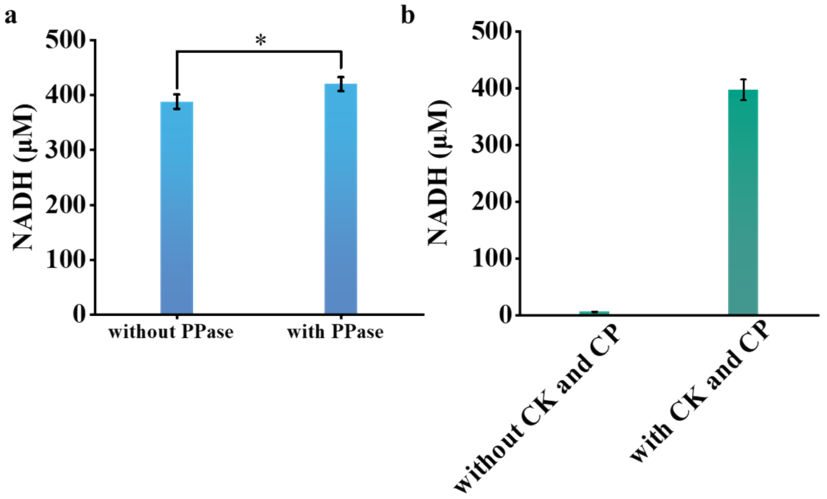

Figure S9. The effect of CK and PPase on NADH production. (a) Concentration of produced NADH in a solution containing RK of 300 U/mL, RPPK of 1800 U/mL, NAMPT of 600 U/mL, NMNAT of 75 U/mL, FDH of 375 U/mL, CK of 60 μg/mL, D-ribose (10 mM), NAM (5 mM), ATP (8 mM), sodium formate (2.4 mM), and sodium creatine phosphate (10 mM) at 37°C in Tris-HCl (pH 8.0) without PPase, and with 50 U/mL PPase. (b) Concentration of NADH in solutions containing RK of 300 U/mL, RPPK of 1800 U/mL, NAMPT of 600 U/mL, NMNAT of 75 U/mL, FDH of 375 U/mL, D-ribose (10 mM), NAM (5 mM), ATP (8 mM), and sodium formate (2.4 mM), at 37°C in Tris-HCl (pH 8.0) with and without CK (60 μg/μL) and CP (10 mM).

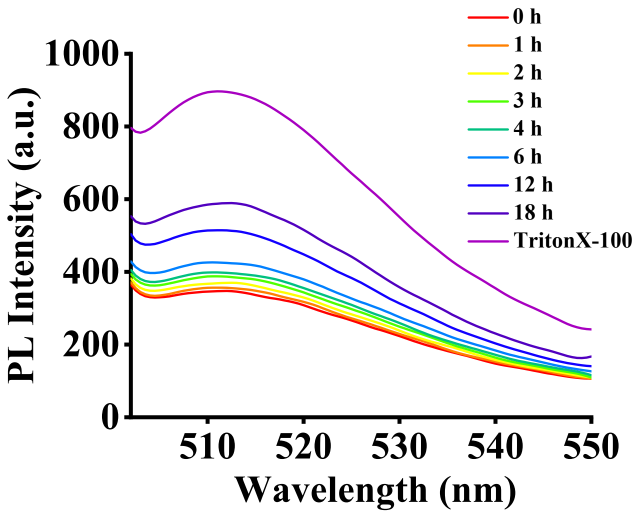

Figure S10. The determination of the leakage rate in GUVs. (a) Fluorescence spectra of the external solution of vesicles containing calcein. The curves from bottom to top correspond to incubation times of 0 h, 1 h, 2 h, 3 h, 4 h, 6 h, 12 h, 18 h, and complete calcein release after the addition of Triton X-100 at 37°C, respectively.

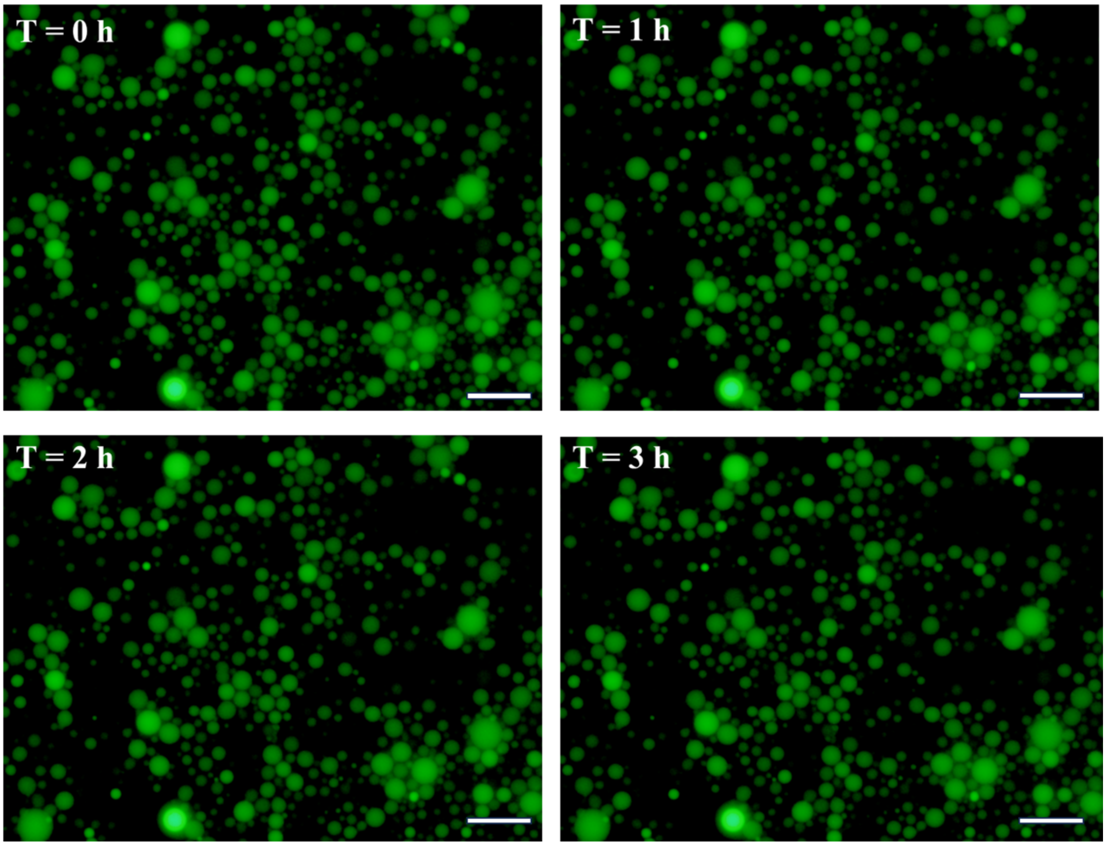

Figure S11. Representative fluorescence microscopy images of GUVs encapsulated calcein after incubation at 37 °C for 0 h, 1 h, 2 h, and 3 h. Scale bars are 50 μm.

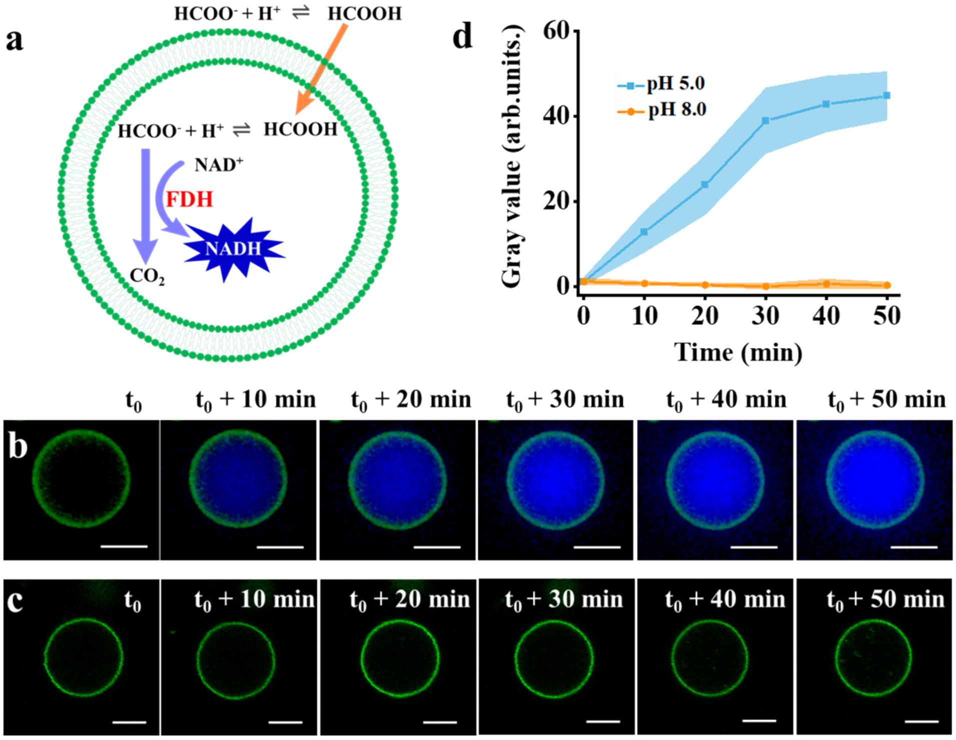

Figure S12. Time-lapse fluorescence images of produced NADH inside artificial cells by adding formate. (a) schematic representation of the metabolic pathway converting NAD^+ to NADH in artificial cells by adding formate. (b) Representative time-lapse images of artificial cells in pH 5.0 external solution after adding sodium formate (2.4 mM). (c) Representative time-lapse images of artificial cells in pH 8.0 external solution after adding sodium formate (2.4 mM). (d) Corresponding fluorescence intensity of artificial cell in (b) and (c). Scale bars are 10 μm.

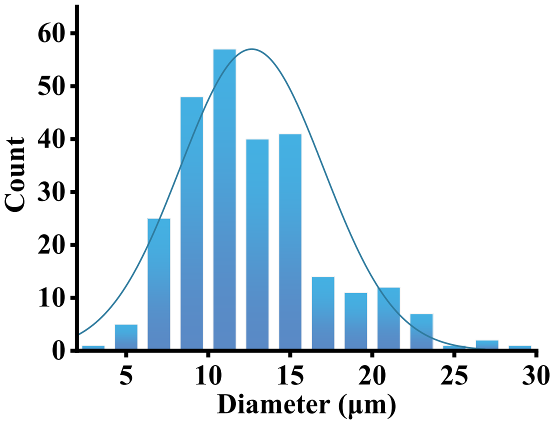

Figure S13. Histogram of GUVs diameters obtained from microscopy images with a sample size of 268.

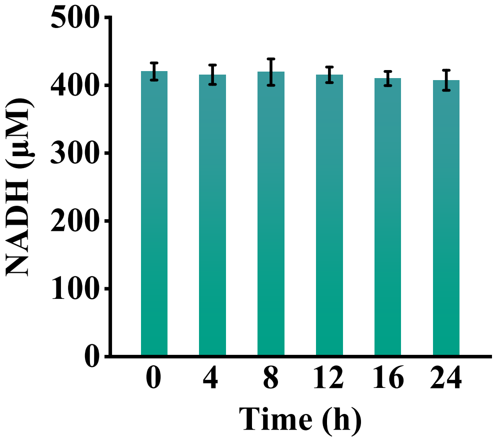

Figure S14. NADH production in artificial cells containing NADH synthesis pathway stored at 4°C as a function of time.

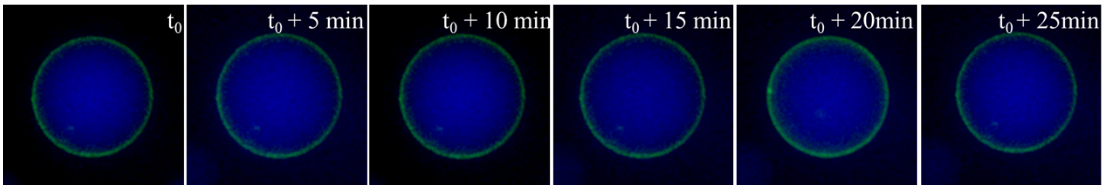

Figure S15. Representative time-lapse fluorescence microscopy images of artificial cells containing NH_4Cl, α-ketoglutarate, and NADH in the absence of GDH.

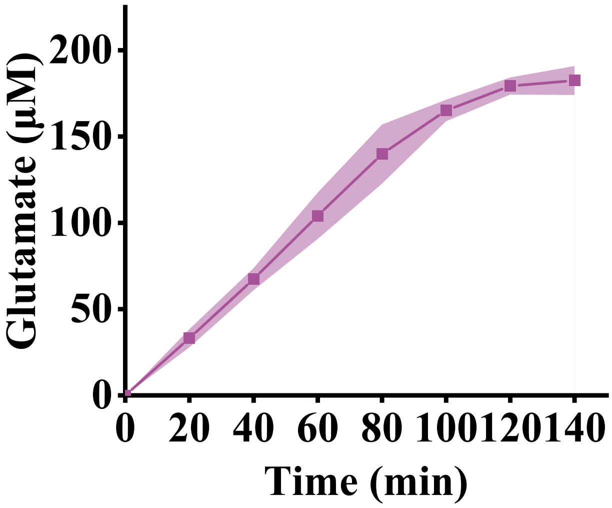

Figure S16. The concentration of produced glutamate against reaction time in solution with RK of 300 U/mL, RPPK of 1800 U/mL, NAMPT of 600 U/mL, NMNAT of 75 U/mL, FDH of 375 U/mL, GDH of 20 U/mL, CK of 60 μg/mL, PPase of 50 U/ml, D-ribose (10 mM), NAM (5 mM), ATP (8 mM), sodium formate (2.4 mM), sodium creatine phosphate (10 mM), NH_4Cl (10 mM) and α-ketoglutarate (0.5 mM) at 37°C in 50 mM Tris-HCl (pH 8.0).

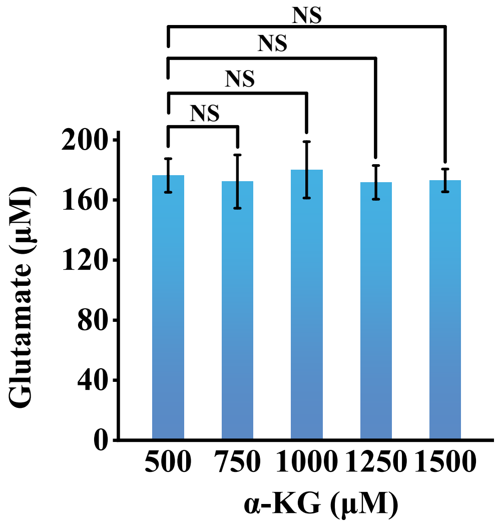

Figure S17. Production of glutamate at concentrations of α-KG of 500, 750, 1000, 1250, and 1500 μM in 50 mM Tris-HCl (pH 8.0).

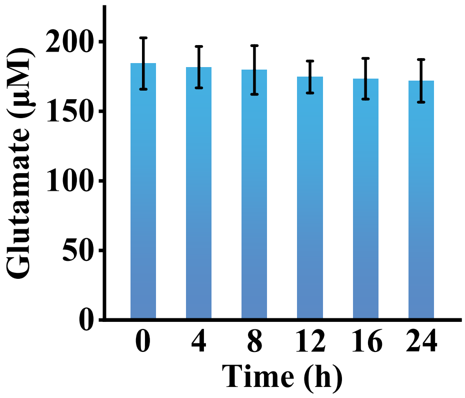

Figure S18. Glutamate production in artificial cells containing glutamate synthesis pathway stored at 4°C as a function of time.

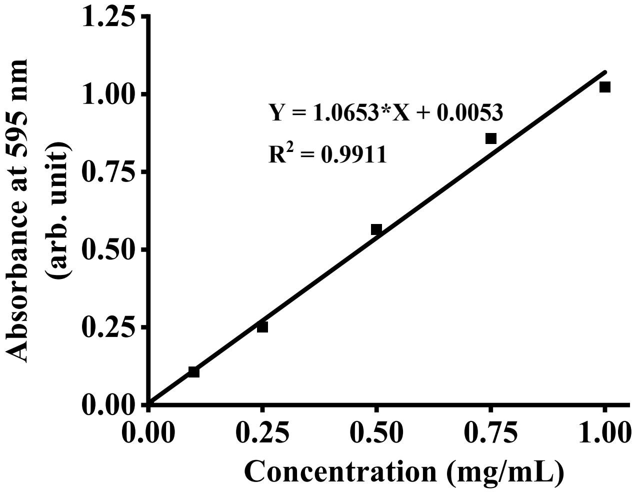

Figure S19. A calibration curve for protein concentration.

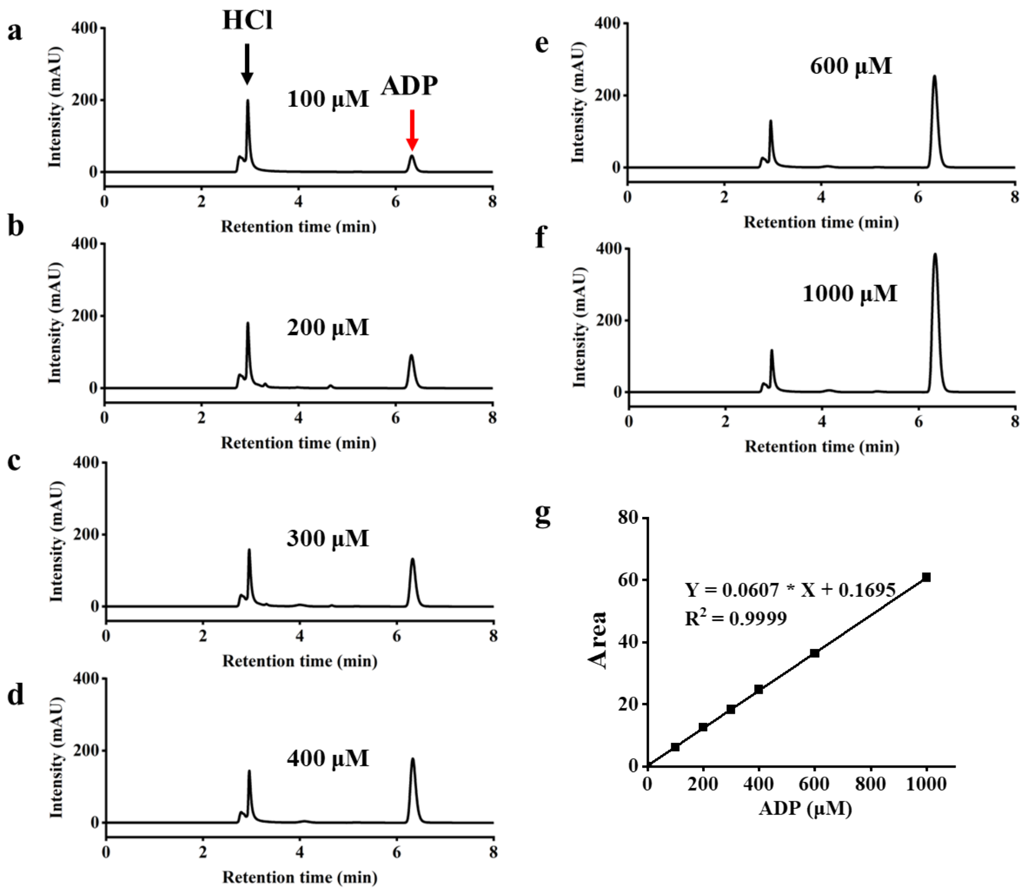

Figure S20. Representative HPLC chromatograms of ADP at concentrations of 100 μM (a), 200 μM (b), 300 μM (c), 400 μM (d), 600 μM (d), and 1000 μM (e), in Tris-HCl buffer. (g) Calibration curve of ADP.

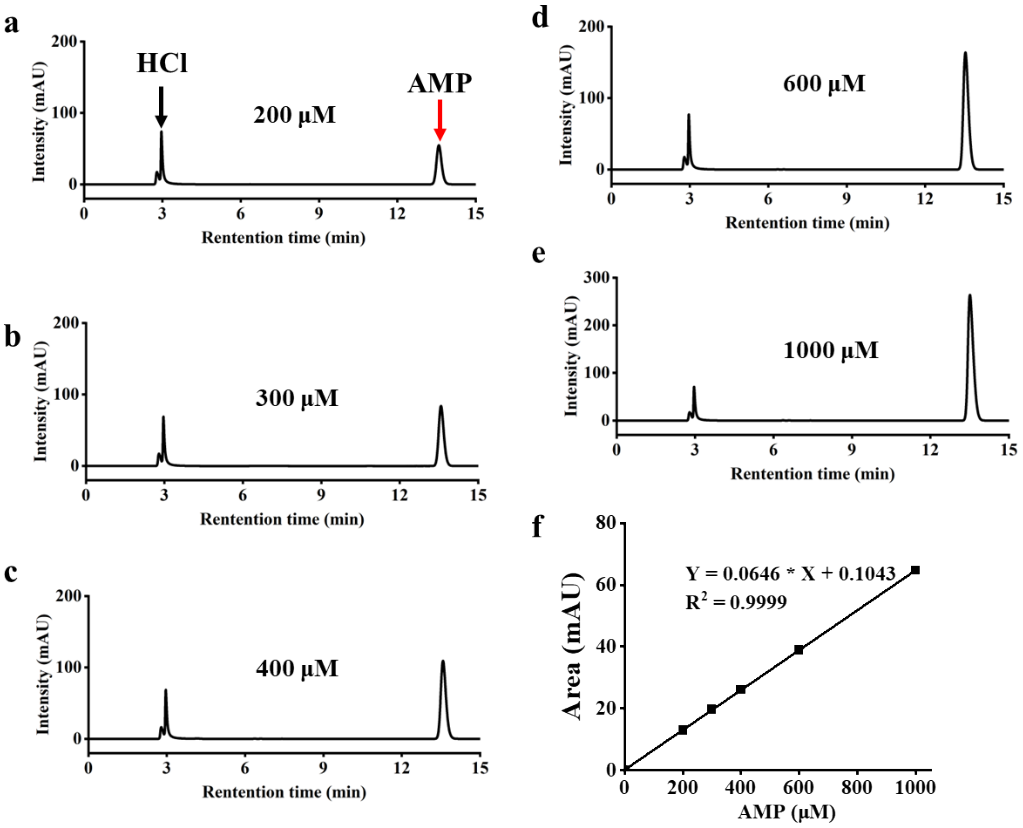

Figure S21. Representative HPLC chromatograms of AMP at concentrations of 200 µM (a), 300 µM (b), 400 µM (c), 600 µM (d), and 1000 µM (e), in Tris-HCl buffer. (f) Calibration curve of AMP.

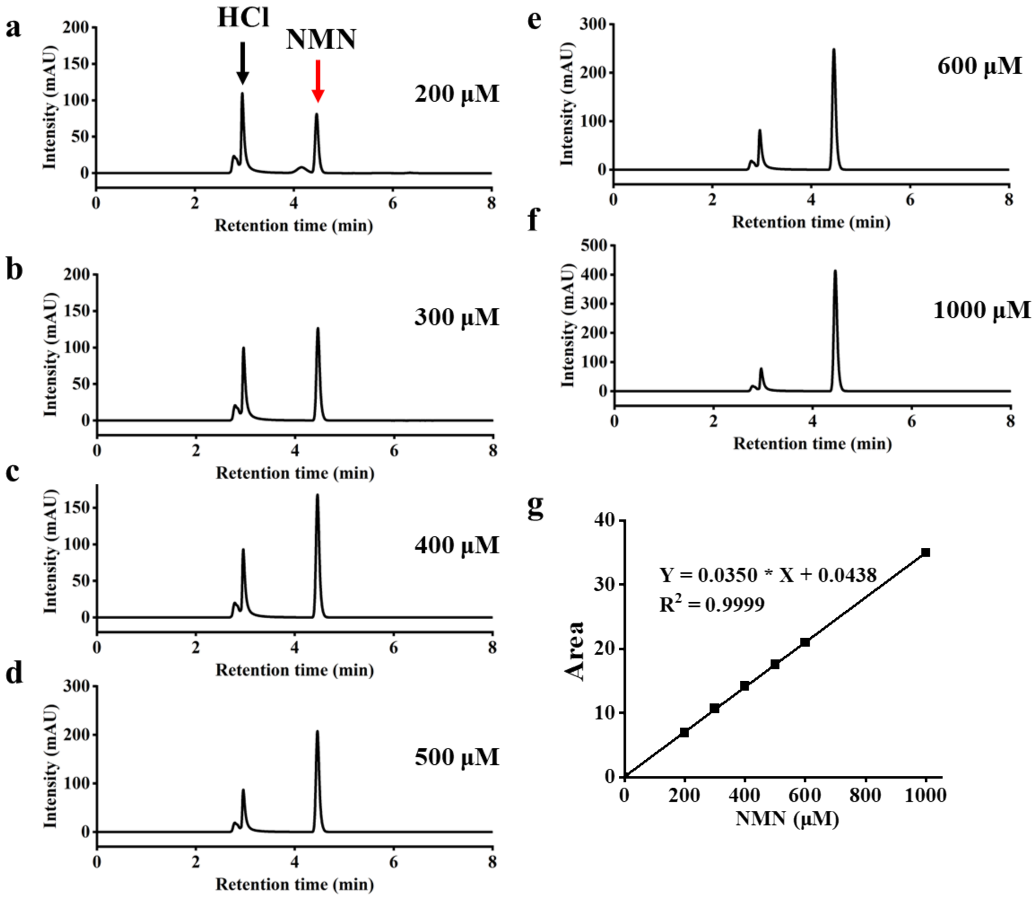

Figure S22. Representative HPLC chromatograms of NMN at concentrations of 200 μM (a), 300 μM (b), 400 μM (c), 500 μM (d), 600 μM (e), and 1000 μM (f), in Tris-HCl buffer. (g) Calibration curve of NMN.

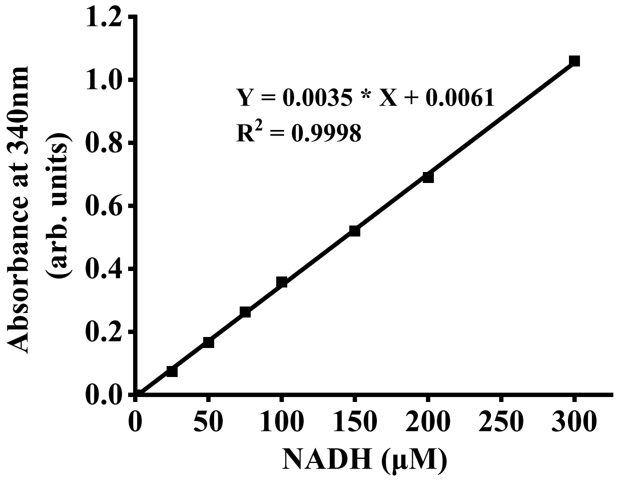

Figure S23. A calibration curve of NADH.

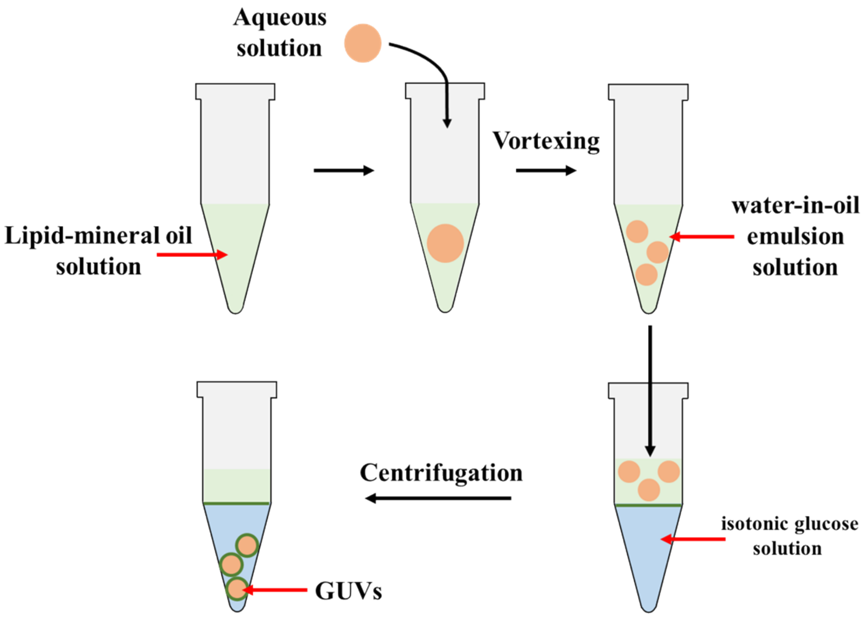

Figure S24. Schematic diagram of vesicle preparation by emulsion method.

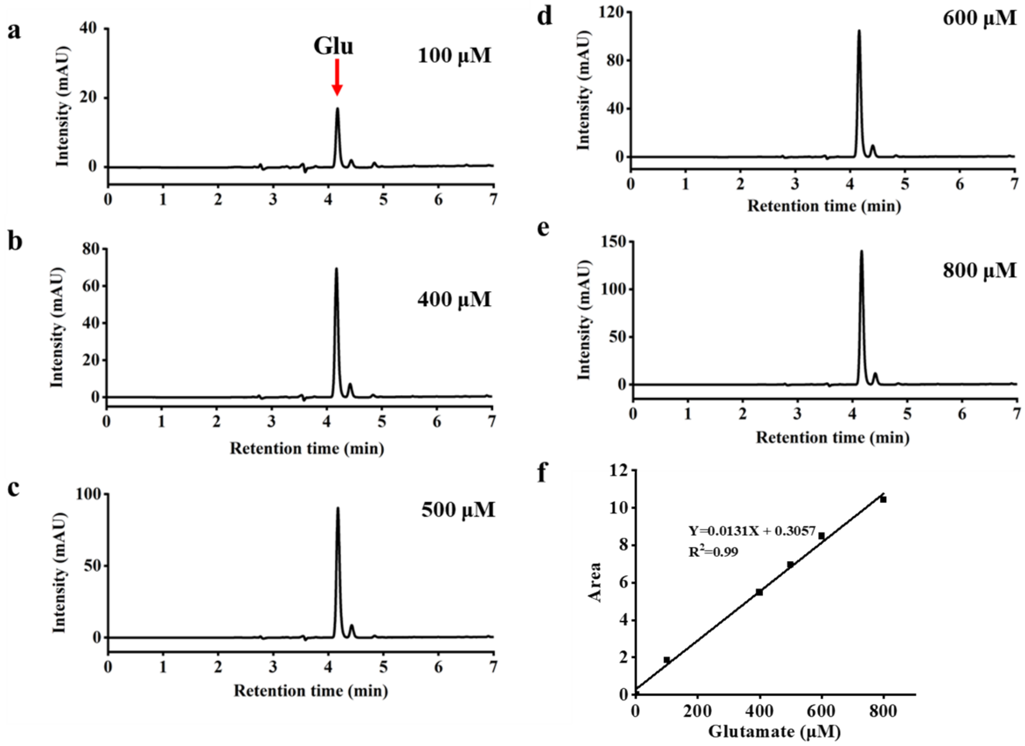

Figure S25. Representative HPLC chromatograms of glutamate at concentrations of 100 μM (a), 400 μM (b), 500 μM (c), 600 μM (d), and 800 μM (e), in Tris-HCl buffer. (f) Calibration curve of glutamate.

Table S1. Primers used for of all plasmid used in this study.

gene	5’	3’
rbsK	ATGCAAAACGCAGGCAGCCT	TCACCTCTGCCTGTCTAAAAATGCGTC
prs	TTGAGCCACGACTGGACCGAT	CTATGCGTCCCCGTCGAAAAGTCCTG
nampt	ATGACTGCACCATCCCAGGT	TTGTTTAACGGACCAGCGTACCGTTTCCCC
nadM	ATGTACAAATTTGATTATCTCGTT	TTAGATTCGAGCTCGATATTTTTGTTG
fdh	ATGTCAGCAAAGCAAGTCTCGAA	TTAGACCCATCCGCGGAAACGCGATG
rocG	ATGTCAGCAAAGCAAGTCTCGAA	TTAGACCCATCCGCGGAAACGCG

Table S2. The DNA sequences of all enzymes used in this study.

Enzymes	Gene symbol	Sequence
RK	rbsK	ATGCAAAACGCAGGCAGCCTCGTTGTTCTTGGCAGCATTAATGCTGACCACATTCTTAATCTTCAATCTTTTCCTACTCCAGGCGAAACCGTAACCGGTAACCACTATCAGGTTGCATTTGGCGGCAAAGGCGCGAATCAGGCTGTGGCTGCTGGGCGTAGCGGTGCGAATATCGCGTTTATTGCCTGTACGGGTGATGACAGCATTGGTGAGAGCGTTCGCCAGCAGCTCGCCACTGATAACATTGATATTACTCCGGTCAGCGTGATCAAAGGCGAATCAACAGGTGTGGCGCTGATTTTTGTTAATGGCGAAGGTGAGAATGTCATCGGTATTCATGCCGGCGCTAATGCTGCCCTTTCCCCGGCGCTGGTGGAAGCGCAACGTGAGCGTATTGCCAACGCGTCAGCATTATTAATGCAGCTGGAATCACCACTCGAAAGTGTGATGGCAGCGGCGAAAATCGCCCATCAAAATAAGACTATCGTTGCGCTTAACCCGGCTCCGGCTCGCGAACTTCCTGACGAACTGCTGGCGCTGGTGGACATTATTACGCCAAACGAAACGGAAGCAGAAAAGCTCACCGGTATTCGTGTTGAAAATGATGAAGATGCAGCGAAGGCGGCGCAGGTACTGCATGAAAAAGGTATCCGTACTGTACTGATTACTTTAGGAAGTCGTGGTGTATGGGCTAGCGTGAATGGTGAAGGTCAGCGCGTTCCTGGATTCCGGGTGCAGGCTGTCGATACCATTGCTGCCGGAGATACCTTTAACGGTGCGTTAATCACGGCATTGCTGGAAGAAAAACCATTGCCAGAGGCGATTCGTTTTGCCCATGCTGCCGCTGCGATTGCCGTAACACGTAAAGGCGCACAACCTTCCGTACCGTGGCGTGAAGAGATCGACGCATTTTTAGACAGGCAGAGGTGA
RPPK	prs	TTGAGCCACGACTGGACCGATAATCGCAAAAACCTGATGCTCTTTGCCGGCCGCGCCCATCCGGAGCTGGCCGAGCAGGTAGCCAAAGAGCTCGACGTCCACGTCACCTCTCAGGACGCGCGGGAGTTCGCCAACGGCGAGATCTTCGTGCGCTTCCACGAATCGGTACGCGGTTGCGACGCCTTCGTCCTGCAATCCTGCCCGGCACCGGTGAACAGGTGGCTGATGGAACAGCTGATCATGATCGACGCGCTCAAACGGGGCAGCGCCAAACGGATCACCGCCGTCATGCCGTTCTATCCGTATGCCCGGCAAGACAAGAAACACCGTGGCCGCGAACCGATCTCCGCGCGACTGATCGCCGACCTGCTCAAGACCGCGGGCGCCGACCGGATCGTGACGGTCGACCTGCACACCGACCAGATCCAGGGTTTCTTCGACGGGCCGGTCGATCATATGCGCGGTCAGAACCTGCTGACCGGTTACATCAGAGACAACTACCCGGACGGCAACATGGTGGTCGTCTCCCCTGACTCCGGCCGGGTACGCATCGCCGAGAAGTGGGCCGACGCATTAGGTGGTGTTCCCC

		TCGCCTTCATCCACAAGACCCGTGATCCGCGGGTACCCAACCAAGT<br>GGTGTCCAACCGCGTCGTCGGCGACGTGGCCGGGCGCACCTGTGT<br>CCTGATTGACGACATGATCGACACCGGCGGCACCATCGCCGGCGC<br>GGTGGCATTGCTGCACAACGACGGCGCCGGTGACGTGATCATCGC<br>GGCAACCCACGGCGTGCTCTCCGACCCCGCTGCGCAGCGGCTGGC<br>CTCCTGCGGCGCCCGCGAAGTGATCGTCACGAACACGCTCCCGAT<br>CGGCGAAGACAAGCGCTTCCCCCAGCTCACCGTTTTGTCCATCGCG<br>CCGCTGCTGGCCAGCACAATCCGGGCGGTCTTCGAAAACGGCTCA<br>GTAACAGGACTTTTCGACGGGGACGCATAG
NAMPT	nampt	ATGACTGCACCATCCCAGGTATTAAAGATCCGCCGCCCAGACGAC<br>TGGCACCTTCACCTCCGCGATGGCGACATGTTAAAAACTGTCGTGC<br>CATATACCAGCGAAATTTATGGACGGGCTATCGTAATGCCCAATCT<br>GGCTCCGCCCGTGACCACCGTTGAGGCTGCCGTGGCGTATCGCCA<br>GCGTATTCTTGACGCCGTACCTGCCGGGCACGATTTCACCCCATTG<br>ATGACCTGTTATTTAACAGATTCGCTGGATCCTAATGAGCTGGAGC<br>GCGGATTTAACGAAGGCGTGTTCACCGCTGCAAAACTTTACCCGG<br>CAAACGCAACCACTAACTCCAGCCACGGCGTGACGTCAATTGACG<br>CAATCATGCCGGTACTTGAGCGCATGGAAAAAATCGGTATGCCGC<br>TACTGGTGCATGGTGAAGTGACACATGCAGATATCGACATTTTTGA<br>TCGTGAAGCGCGCTTTATAGAAAGCGTGATGGAACCTCTGCGCCA<br>GCGCCTGACTGCGCTGAAAGTCGTTTTTGAGCACATCACCACCAAA<br>GATGCTGCCGACTATGTCCGTGACGGAAATGAACGGCTGGCTGCC<br>ACCATCACTCCGCAGCATCTGATGTTTAACCGCAACCATATGCTGG<br>TTGGAGGCGTGCGTCCGCACCTGTATTGTCTACCCATCCTCAAACG<br>TAATATTCACCAACAGGCATTGCGTGAACTGGTCGCCAGCGGTTTT<br>AATCGAGTATTCCTCGGTACGGATTCTGCGCCACATGCACGTCATC<br>GCAAAGAGAGCAGTTGCGGCTGCGCGGGCTGCTTCAACGCCCCAA<br>CCGCGCTGGGCAGTTACGCTACCGTCTTTGAAGAAATGAATGCTTT<br>GCAGCACTTTGAAGCATTCTGTTCTGTAAACGGCCCGCAGTTCTAT<br>GGGTTGCCGGTCAACGACACATTCATCGAACTGGTACGTGAAGAG<br>CAACAGGTTGCTGAAAGCATCGCACTGACTGATGACACGCTGGTG<br>CCATTCCTCGCCGGGGAAACGGTACGCTGGTCCGTTAAACAATAA
NMNAT	nadM	ATGTACAAATTTGATTATCTCGTTTTTATTGGGCGCTTTCAGCCGTT<br>TCATTTCGCACATTTGCAGACTATACAAATTGCATTACAGCAAAGT<br>CGTGAGGTTATTATTGCATTGGGATCTGCTCAACCTGAGCGTAATA<br>TTAAGAATCCATTTCTTGCGGAAGAACGTCAAAAAATGATTCTGGC<br>AAATTTTTCAGCAGAAGATCAGGCTCGGATTCATTTTGTAAATATT<br>ATTGATGTTTATAATGATCAGAAATGGGTAGAGCAGGTTAAGCAA

		TTGGTCAACGCTATAATTGAATCAAGAAGTCATGTTGGCTTGATTG<br>GACATTTTAAAGATGAATCTTCATATTATCTAAAACTTTTTCCAGA<br>ATGGACTATGGTCGAACTAGAAAGTTTAAAAGAGTCGATGTCAGC<br>AACACCAATGCGAGAAGCTTACTATGAAGGTAAAATCATTGAGTC<br>GGCATTTCCAGAAGGTACAATTCAGTTTTTAAAAACATTTCAAGAT<br>TCTGAAATTTATAAACAACTACAACAAAAATATCGAGCTCAAGAT<br>TCATCAAATTTGATCTAA
FDH	fdh	ATGTCAGCAAAGCAAGTCTCGAAAGATGAAGAAAAAGAAGCTCTT<br>AACTTATTTCTGTCTACCCAAACAATCATTAAGGAAGCCCTTCGGA<br>AGCTGGGTTATCCGGGAGATATGTATGAACTCATGAAAGAGCCGC<br>AGAGAATGCTCACTGTCCGCATTCCGGTCAAAATGGACAATGGGA<br>GCGTCAAAGTGTTCACAGGCTACCGGTCACAGCACAATGATGCTG<br>TCGGTCCGACAAAGGGGGGCGTTCGCTTCCATCCAGAAGTTAATG<br>AAGAGGAAGTAAAGGCATTATCCATTTGGATGACGCTCAAATGCG<br>GGATTGCCAATCTTCCTTACGGCGGCGGGAAGGGCGGTATTATTTG<br>TGATCCGCGGACAATGTCATTTGGAGAACTGGAAAGGCTGAGCAG<br>GGGGTATGTCCGTGCCATCAGCCAGATCGTCGGTCCGACAAAGGA<br>TATTCCAGCTCCCGATGTGTACACCAATTCGCAGATTATGGCGTGG<br>ATGATGGATGAGTACAGCCGGCTGCGGGAATTCGATTCTCCGGGC<br>TTTATTACAGGTAAACCGCTTGTTTTGGGAGGATCGCAAGGACGG<br>GAAACAGCGACGGCACAGGGCGTCACGATTTGTATTGAAGAGGCG<br>GTGAAGAAAAAAGGGATCAAGCTGCAAAACGCGCGCATCATCATA<br>CAGGGCTTTGGAAACGCGGGTAGCTTCCTGGCCAAATTCATGCAC<br>GATGCGGGCGCGAAGGTGATCGGGATTTCTGATGCCAATGGCGGG<br>CTCTACAACCCAGACGGCCTTGATATCCCTTATTTGCTCGATAAAC<br>GGGACAGCTTTGGTATGGTCACCAATTTATTTACTGACGTCATCAC<br>AAATGAGGAGCTGCTTGAAAAGGATTGCGATATTTTAGTGCCTGC<br>CGCGATCTCCAATCAAATCACAGCCAAAAACGCACATAACATTCA<br>GGCGTCAATCGTCGTTGAAGCGGCGAACGGCCCGACAACCATTGA<br>TGCCACTAAGATCCTGAATGAAAGAGGCGTGCTGCTTGTGCCGGA<br>TATCCTAGCGAGTGCCGGCGGCGTCACGGTTTCTTATTTTGAATGG<br>GTGCAAAACAACCAAGGATATTATTGGTCGGAAGAAGAGGTTGCA<br>GAAAAACTGAGAAGCGTCATGGTCAGCTCGTTCGAAACAATTTAT<br>CAAACAGCGGCAACACATAAAGTGGATATGCGTTTGGCGGCTTAC<br>ATGACGGGCATCAGAAAATCGGCAGAAGCATCGCGTTTCCGCGGA<br>TGGGTCTAA
GDH	rocG	ATGTCAGCAAAGCAAGTCTCGAAAGATGAAGAAAAAGAAGCTCTT<br>AACTTATTTCTGTCTACCCAAACAATCATTAAGGAAGCCCTTCGGA

		AGCTGGGTTATCCGGGAGATATGTATGAACTCATGAAAGAGCCGC<br>AGAGAATGCTCACTGTCCGCATTCCGGTCAAAATGGACAATGGGA<br>GCGTCAAAGTGTTCACAGGCTACCGGTCACAGCACAATGATGCTG<br>TCGGTCCGACAAAGGGGGGCGTTCGCTTCCATCCAGAAGTTAATG<br>AAGAGGAAGTAAAGGCATTATCCATTTGGATGACGCTCAAATGCG<br>GGATTGCCAATCTTCCTTACGGCGGCGGGAAGGGCGGTATTATTTG<br>TGATCCGCGGACAATGTCATTTGGAGAACTGGAAAGGCTGAGCAG<br>GGGGTATGTCCGTGCCATCAGCCAGATCGTCGGTCCGACAAAGGA<br>TATTCCAGCTCCCGATGTGTACACCAATTCGCAGATTATGGCGTGG<br>ATGATGGATGAGTACAGCCGGCTGCGGGAATTCGATTCTCCGGGC<br>TTTATTACAGGTAAACCGCTTGTTTTGGGAGGATCGCAAGGACGG<br>GAAACAGCGACGGCACAGGGCGTCACGATTTGTATTGAAGAGGCG<br>GTGAAGAAAAAAGGGATCAAGCTGCAAAACGCGCGCATCATCATA<br>CAGGGCTTTGGAAACGCGGGTAGCTTCCTGGCCAAATTCATGCAC<br>GATGCGGGCGCGAAGGTGATCGGGATTTCTGATGCCAATGGCGGG<br>CTCTACAACCCAGACGGCCTTGATATCCCTTATTTGCTCGATAAAC<br>GGGACAGCTTTGGTATGGTCACCAATTTATTTACTGACGTCATCAC<br>AAATGAGGAGCTGCTTGAAAAGGATTGCGATATTTTAGTGCCTGC<br>CGCGATCTCCAATCAAATCACAGCCAAAAACGCACATAACATTCA<br>GGCGTCAATCGTCGTTGAAGCGGCGAACGGCCCGACAACCATTGA<br>TGCCACTAAGATCCTGAATGAAAGAGGCGTGCTGCTTGTGCCGGA<br>TATCCTAGCGAGTGCCGGCGGCGTCACGGTTTCTTATTTTGAATGG<br>GTGCAAAACAACCAAGGATATTATTGGTCGGAAGAAGAGGTTGCA<br>GAAAAACTGAGAAGCGTCATGGTCAGCTCGTTCGAAACAATTTAT<br>CAAACAGCGGCAACACATAAAGTGGATATGCGTTTGGCGGCTTAC<br>ATGACGGGCATCAGAAAATCGGCAGAAGCATCGCGTTTCCGCGGA<br>TGGGTCTAA
